# Current state of research on acupuncture for the treatment of post-stroke dysphagia: a scoping review

**DOI:** 10.3389/fnins.2024.1391576

**Published:** 2024-08-15

**Authors:** Haoran Guo, Xingfang Pan, Yujie Zheng, Xue Yang, Hanyu Xu, Yuan Zhang, Yuqi Sun, Zeran Wang, Te Ba, Bo Pang, Ting Hao, Junhua Zhang, Xiaofeng Zhao

**Affiliations:** ^1^Department of Acupuncture and Moxibustion, First Teaching Hospital of Tianjin University of Traditional Chinese Medicine, Tianjin, China; ^2^National Clinical Research Center for Chinese Medicine Acupuncture and Moxibustion, Tianjin, China; ^3^Research Center of Experimental Acupuncture Science, Tianjin University of Traditional Chinese Medicine, Tianjin, China; ^4^School of Acupuncture and Moxibustion and Tuina, Tianjin University of Traditional Chinese Medicine, Tianjin, China; ^5^Evidence-Based Medicine Center, Tianjin University of Traditional Chinese Medicine, Tianjin, China

**Keywords:** acupuncture, electroacupuncture, post-stroke dysphagia, scoping review, clinical studies

## Abstract

**Objective:**

Post-stroke dysphagia (PSD) is a common complication of stroke. Acupuncture as one of the traditional therapies in traditional Chinese medicine (TCM), can change the excitability of cerebral cortical nerve cells, and promote the recovery of neurological and swallowing functions. Several clinical primary studies (including RCTs, cohort studies, etc.) and systematic reviews have demonstrated its efficacy and safety in patients with PSD. The positive effects of acupuncture on PSD are also mentioned in international clinical and treatment guidelines, while there is no synthesis of this evidence. This scoping review aims to summarize the evidence from clinical primary studies, reviews, systematic reviews, and guidelines on acupuncture for the treatment of PSD and explore the breadth of this evidence, provide an overview of the range and characteristics of existing evidence, research gaps, and future research priorities in treating PSD with acupuncture.

**Method:**

PubMed, Embase, Cochrane Library, Web of Science, China National Knowledge Infrastructure, SinoMed, Wan Fang Data, and VIP databases were searched from inception until June 12, 2024. The relevant data were presented through bubble diagrams, line graphs, and structured tables along with descriptive statistics and analysis. This scoping review was conducted based on the PRISMA-ScR Checklist.

**Results:**

A total of 1,130 studies were included. Most of the studies were conducted in China, with the number increasing over time. The studies included 254 reviews, 815 clinical studies (678 RCTs,107 nRCTs, 12 case reports, 14 cohort studies, and four case series), 51 systematic reviews, and 10 guidelines. Acupuncture interventions included manual acupuncture (MA), electroacupuncture (EA), and MA/EA combined with acupuncture-related methods (such as scalp acupuncture, auricular acupuncture, warm acupuncture, etc.). The most frequently used acupoint was RN23. Acupuncture is often applied in combination with other treatments, such as herbal medicine, Western medicine, rehabilitation training, swallowing training, or catheter balloon dilatation. Effective rates and WTS were the most frequently used outcomes. Most studies reported significant efficacy and only a few studies explicitly reported adverse events. Acupuncture received positive recommendations in nine guidelines for the treatment of PSD.

**Conclusion:**

As a convenient and safe traditional Chinese medicine therapy with its characteristics, acupuncture can improve different stages and types of dysphagia without causing serious adverse reactions. In the future, more standardized international cooperative clinical research is needed to identify the influence of different acupuncture intervention times on the curative effect and dose-effect relationship of acupuncture; standardize the clinical acupoint selection scheme of acupuncture; develop a COS with TCM characteristics to improve the quality of outcome reporting, This will enable different research data to be summarized and compared, reduce resource waste, and provide more high-quality evidence.

## 1 Introduction

Stroke is considered one of the leading causes of adult mortality and disability (GBD 2019 Stroke Collaborators, 2021), with over 12.2 million new strokes each year. Globally, one in four people over age 25 will have a stroke in their lifetime (Feigin et al., 2022). Dysphagia is one of the most common complications of stroke, with an estimated prevalence ranging from 30% to 80% (Sun et al., 2023). The occurrence of post-stroke dysphagia is associated with dysfunction of certain organs such as the lips, tongue, and pharynx, caused by damage to the swallowing cortex, cortical medullary damage, and damage to the medullary swallowing center (Zhu et al., 2023). The main clinical manifestations are choking and coughing while drinking water, dysphagia, and hoarseness. Dysphagia can lead to aspiration pneumonia, asphyxia, malnutrition, dehydration, psychological disorders, chest infections, or even death (Wang Y. et al., 2023). These complications can further aggravate dysphagia, seriously affect the quality of life and rehabilitation process of patients, prolong hospital stays, increase medical expenses, and impose a significant economic burden on individuals and society (Xu et al., 2023). At present, many clinical methods have been used to treat dysphagia, such as swallowing training, noninvasive transcranial direct current stimulation, neuromuscular electrical stimulation, cold stimulation, biofeedback therapy, and balloon dilatation therapy (Li et al., 2023). However, there is still no specific and effective treatment for post-stroke dysphagia. As a traditional peripheral superficial stimulation method, acupuncture has been widely used in routine clinical practice for post-stroke dysphagia and has achieved a good curative effect (Jiang et al., 2022; Li et al., 2023).

The 2016 American Stroke Association (ASA) guidelines (Winstein et al., 2016) mentioned that acupuncture may be a beneficial alternative treatment for dysphagia. The 2021 European guidelines for the diagnosis and treatment of PSD (Dziewas et al., 2021) suggested that acupuncture may be used to rehabilitate swallowing function. Many RCTs and systematic reviews of acupuncture for PSD have been published, showing that acupuncture can re-establish swallowing function and effectively improve the quality of life of PSD patients without serious adverse events (Zhong et al., 2021; Zhang et al., 2024). In addition to efficacy and safety, several questions remain to be addressed in clinical studies of acupuncture for PSD. To provide an overview of the range and characteristics of existing evidence, research gaps, and future research priorities in treating PSD with acupuncture, we conducted a scoping review to summarize and critically analyze the findings of all published articles (Munn et al., 2018).

The scoping review is an approach for synthesizing research evidence. Scoping reviews are particularly helpful when the literature is complex and heterogeneous, as they can identify published studies, reducing wasted resources, duplication, and research waste. The most common reasons for conducting scoping reviews are to explore the breadth or depth of the literature, map and summarize the evidence, and identify areas for future systematic reviews or other types of evidence synthesis. This can provide information about potential limitations of future work and inform approaches, potentially saving time and resources (Peters et al., 2020; Campbell et al., 2023). We conducted this scoping review to describe and synthesize previous studies on acupuncture for PSD, and to identify evidence gaps and future research needs in this area.

## 2 Methods

This scoping review follows the PRISMA Extension for Scoping Reviews (PRISMA-ScR) (Page et al., 2021). We reviewed the literature in accordance with Arksey and O'Malley's methodological approach for scoping reviews through the following five stages: (a) identification of the research questions, (b) identification of the relevant studies, (c) selection of studies for review, (d) extraction and charting of the data, and (e) summary of the results.

### 2.1 Identifying the review questions

Before starting this study, the broad exploratory research question was, “What has been studied about acupuncture treatments administered to patients with PSD?” The more detailed research questions were as follows: what kind of clinical research has been conducted? What are the characteristics of acupuncture interventions in clinical research? Which indicators are used to evaluate the effectiveness and safety of the evidence? What are possible directions for future research?

### 2.2 Eligibility criteria

The retrieval strategy followed the principle of PICO. Population: patients with PSD; animal and cell studies were excluded. Intervention: manual acupuncture and electroacupuncture (the term acupuncture refers to puncturing with a needle), studies that used acupuncture alone or in combination with other therapies were also included. Study design: all types of clinical studies were included, including case reports, case series, cohort studies, non-randomized controlled trials (nRCTs), randomized controlled trials (RCTs), and systematic reviews (SRs). No date limitations were applied (inception to June 12, 2024). Language: Chinese or English. Conference articles, abstracts, repeatedly published articles, articles unable to obtain full text, clinical studies with unclear diagnostic criteria, clinical studies with incomplete protocol information (such as lack of selected acupoint, grouping method, outcome indicator, etc.), and studies with incomplete data statistics results were excluded.

### 2.3 Literature search

We searched PubMed, Embase, Cochrane Library, Web of Science (WOS), China National Knowledge Infrastructure (CNKI), VIP Chinese Journal Service Platform (VIP), WanFang Data Knowledge Service Platform (WanFang), and SinoMed databases from inception until June 12, 2024. (In addition, manual searching of the references in this paper was also included.) For each database, we developed an adequate research string that combined the terms “dysphagia”, “swallowing disorders”, “Deglutition disorders”, and “pseudobulbar palsy” with “acupuncture”, “electroacupuncture”, and “stroke”, “stroke cerebral”, “brain infarction”, “cerebral infarction”, “intracranial hemorrhage”, “cerebrovascular accident”, “cerebral hemorrhage”, and searched within titles, abstracts, and keywords. The detailed search strategy adapted for different databases is shown in Supplementary Table S1.

### 2.4 Literature selection and data extraction

All the retrieved articles were imported into NoteExpress. A duplication checking function was used to remove duplicate studies. Two researchers screened the titles and abstracts of the articles independently, and those that met the inclusion criteria were retained for further screening. Subsequently, two researchers read the full text of the articles and retained those that met the criteria. When they disagreed on whether to include a certain article, the two researchers discussed it before making the final decision. If no agreement could be reached, a third researcher would help evaluate whether the article should be included.

The data extraction form consisted of the following information: (1) general information of all studies: title, first author, year of publication, country of origin, study design, type of intervention and control, outcome measures, adverse events, and results; (2) for primary studies: sample size, demographic characteristics of participants (i.e., age, disease course, stage), description of the intervention (i.e., acupuncture type, timing of acupuncture, duration and frequency of intervention, acupoints); (3) for systematic reviews: number of studies, evidence quality evaluation tools, and main conclusions. The Excel table used for extracting the data was designed in advance. Nine researchers worked independently and checked the data in real time to ensure the accuracy of the information.

### 2.5 Analyzing and reporting of the results

After the information from the included studies was collated into tables, the extracts were systematically analyzed. The majority of the data were descriptively evaluated by computing frequencies and percentages, and the outcomes were displayed as structured tables, bubble diagrams, and line graphs. Descriptive information was used to present qualitative data.

## 3 Results

### 3.1 Results of the search

**Figure 1:**
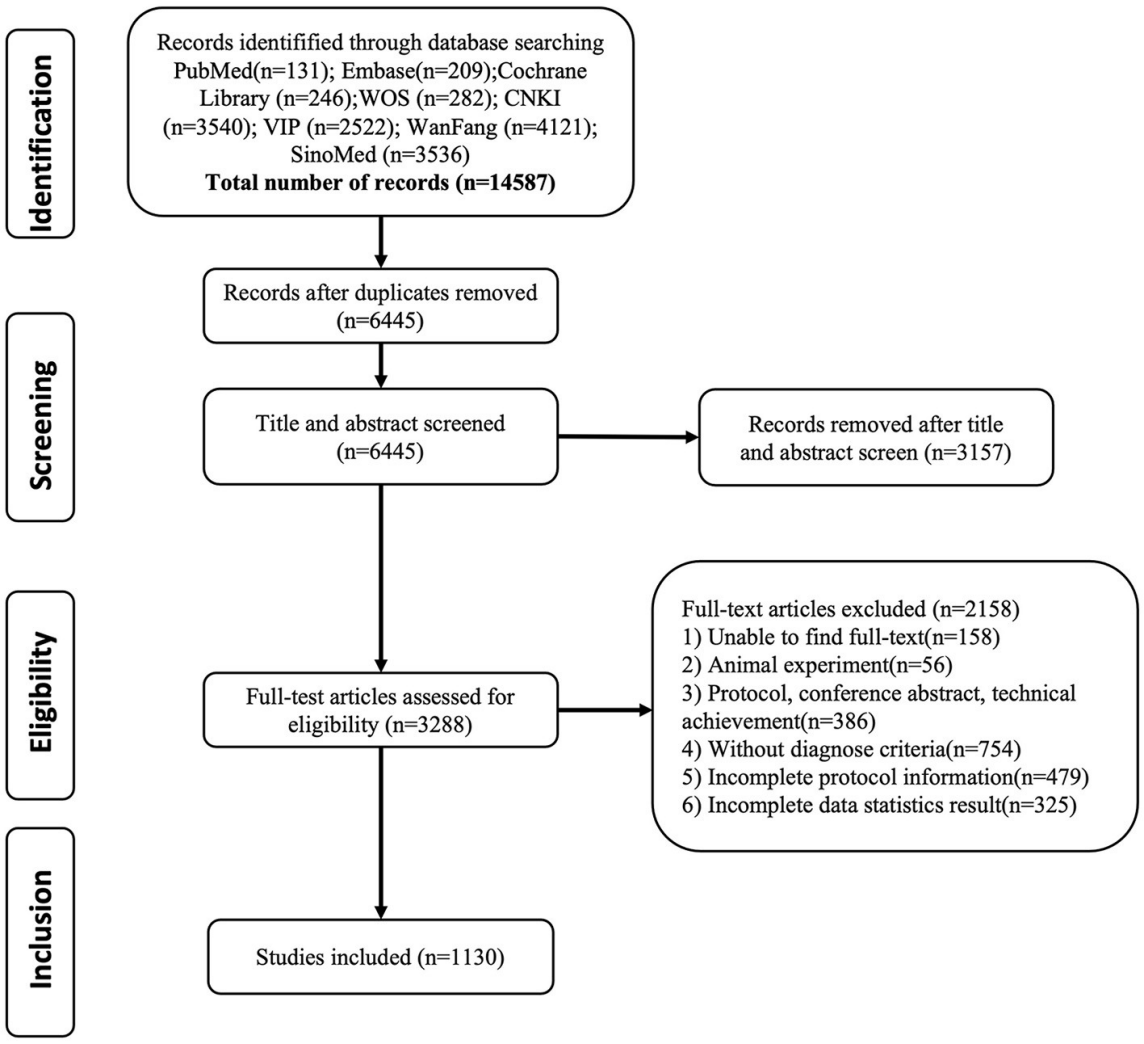
Diagram for scoping review literature identification.

A total of 14,587 articles were retrieved, and 1,130 articles were retained for analysis, including 254 reviews, 815 clinical studies (678 RCTs, 107 nRCTs, 12 case reports, 14 cohort studies, and 4 case series), 51 systematic reviews, and 10 guidelines (Figure 1). There were 188 dissertations among the 1,130 studies. There were 942 journal articles, among which 43 were published in Chinese Acupuncture and Moxibustion, 35 in Shanghai Acupuncture and Moxibustion, 35 in Clinical Journal of Acupuncture and Moxibustion, 19 in Journal of Practical Traditional Chinese Medicine, 16 in Chinese Journal of Physical Medicine and Rehabilitation, 16 in Journal of Emergency in Traditional Chinese Medicine.

### 3.2 Year of publication

**Figure 2:**
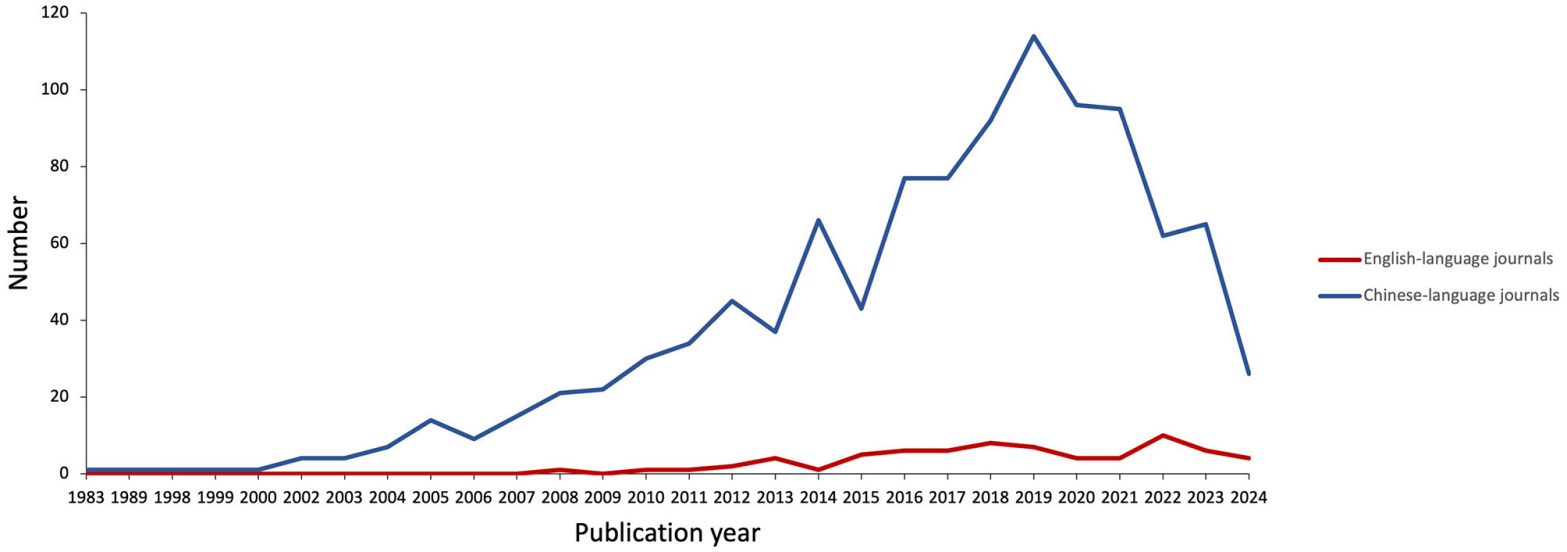
Year of publication of included studies.

The included studies were published from 1983 to June 12, 2024. A total of 1,130 studies were conducted during this period, with 70 published in English and 1,060 in Chinese. The overall number of publications generally showed an upward trend, with the peak number in 2019. The relationship between the volume of literature and publication time is shown in Figure 2.

### 3.3 Consensus on the etiological and therapeutic mechanisms

The 254 reviews covered a wide range of topics, such as famous scholars' experience, evidence in literature, data mining, mechanism of action, clinical application, and research progress.

In traditional Chinese medicine, post-stroke dysphagia can be classified into “she jian” (which means sluggish tongue impeding speech) and “yin fei” (which means the tongue is paralyzed and cannot work well) (Wang X. et al., 2023). Their main clinical manifestations are slow rotation of the tongue, uncontrolled eating, and loss of speech. It is believed in TCM that the etiology and pathogenesis of dysphagia are pathogenic wind, fire, phlegm, blood stasis, and qi deficiency, leading to the dysfunction of Zang-fu organs, reverse flow of qi and blood, obstruction of meridians and collaterals by blood stasis, and oppression of the brain marrow (Tang et al., 2024). The location of sickness is related to the brain, mouth, tongue, and throat. Hence, acupuncture can be used at the corresponding points to nourish yin, activate collaterals, wake up the brain, open the aperture, and remove obstruction (Zhong et al., 2021). Acupuncture is an effective and internationally recognized treatment of stroke that can significantly reduce the disability rate. The selected reviews focused on the therapeutic effect of acupuncture therapy in PSD, as well as treatment thoughts and mechanisms of action.

- The basis of acupoint selection in different stages of swallowing: it has been summarized that the acupoints of acupuncture for dysphagia after stroke are mainly from the head, face, and neck, accounting for 71.54% of all acupoints (Gong, 2020). This area is consistent with the clinical symptoms of PSD patients, indicating that acupuncturists tend to consistently select acupoint locations. Rarely acupoints in the chest, abdomen, and back, possibly because of disease specificity. In a data mining study, the core points identified were GB20, RN23, EX-HN14, Gongxue, MS6, SJ17, EX-HN12, and EX-HN13, with the principal combinations being EX-HN12, EX-HN13, GB20, and RN23 (Wu et al., 2024). Acupuncture at the acupoints on the head, face, and neck is an effective method for the treatment of dysphagia after stroke. These acupoints are rich in nerves, blood vessels, and throat muscles, which can directly or indirectly contact the brain and throat. It can promote the functional repair of neurons in brain tissue (Li et al., 2019); improve cerebral blood flow (Ke et al., 2023); enhance the function of swallowing-related muscles and nerves, help to restore and reconstruct normal reflex arcs; and promote the formation of new central to pharyngeal motor conduction pathways (Chen et al., 2022). To improve dysphagia after stroke. In clinical practice, we should pay attention to the integrated treatment plan of regulating the mind and activating the oral-cavity machine: the head and neck acupoints (such as the middle and lower 1/3 of the motor area, the first, second, and third speech areas, GB20, SJ17, and GB12) should be taken to restore the normal regulation of the cerebral cortex on the cortico-brainstem tract or to restore the function of swallowing reflex arc, so as to improve swallowing function from the center part (Li et al., 2021). The acupoints on the face and mouth (such as ST4, ST6, ST7, SI18, EX-HN10) are taken to improve swallowing function in the oral preparation period and reduce the oral passage time by stimulating the orbicularis oris, buccinator, masseter, medial pterygoid, lateral pterygoid, temporalis, etc. The sublingual acupoints (such as RN23, tri-tongue acupuncture therapy) are selected to promote the recovery of the movement of mylohyoid, digastric, stylohyoid, and other related muscle groups in the oral push stage. The pharyngeal acupoints (such as Tunyan and Tiyan) are selected to promote the recovery of the voluntary movement of constrictor naris, thyrohyoid, pharyngeal levator muscle, and other pharyngeal muscle groups in the pharyngeal stage, and promote the reconstruction of swallowing movements (Cui and Zhang, 2020). Clinical acupoint selection based on the etiology and pathogenesis of the disease and combined with clinical symptoms is carried out, and the prescription of acupoint differentiation is often based on local acupoint selection + distal acupoint selection.
- Mechanism of action: The mechanism of action of acupuncture in the treatment of diseases involves many aspects. Common ones include neurotransmitter regulation, immune system regulation, neuroendocrine regulation, inflammatory factors or cytokine signaling, etc. (Ju et al., 2019). In the treatment of diseases, acupuncture can improve the excitability of nerve cells in the cerebral cortex, to quickly compensate or restore the function of the cerebral cortex. Studies have found that acupuncture can promote the establishment of blood supply and collateral circulation in the ischemic penumbra at the infarct area or the compression edge of edema (Chen et al., 2022; Zhang J. et al., 2023), increase the maximum peak flow velocity (Vs) and average flow velocity (Vm) of bilateral cerebral arteries, and effectively improve the intracranial blood flow in patients with dysphagia after stroke (Hu et al., 2018). Reduce the content of thromboxane B2, angiotensin II, homocysteine, soluble vascular cell adhesion molecule-1, and endothelin-1, increase the content of nitric oxide and vascular endothelial growth factor (VEGF), inhibit platelet aggregation and thrombosis (Tang et al., 2024). Increase the content of serum neurotrophic factors (BDNF, NGF) (Xing et al., 2019). Reducing serum inflammatory factors (such as high-sensitivity C-reactive protein, tumor necrosis factor-α, interleukin-6) in patients with stroke (Zhao et al., 2020). In conclusion, acupuncture has a certain therapeutic effect on patients with dysphagia through a series of mechanisms, such as changing the content of neurotrophic factors, regulating vasomotor factors, vascular cell adhesion molecules, inflammatory factors, blood flow, and improving the excitability of cerebral cortex.

### 3.4 Characteristics of included clinical studies

A total of 815 clinical primary studies were identified in this scoping review, and their characteristics are summarized in Table 1.

**Table 1:** Characteristics of the included clinical studies.

**Characteristics**	**Studies no**.	**(%)**
**Study sample size (*****n*** = **815)**		
≤ 50	90	11.04%
50 <*n* ≤ 100	549	67.36%
100 <*n* ≤ 300	170	20.86%
*n* > 300	6	0.74%
**Participants**		
**Mean age (*****n*** = **505)**		
<55	67	13.27%
55–60	119	23.56%
60–65	198	39.21%
65–70	108	21.39%
>70	13	2.57%
**Mean duration of PSD (*****n*** = **511)**		
≤ 2 weeks	92	18.00%
2–4 weeks	131	25.64%
4–6 weeks	124	24.27%
6–8 weeks	63	12.33%
8–10 weeks	41	8.02%
>10 weeks	60	11.74%
**Stroke type (*****n*** = **502)**		
Ischemic stroke	135	26.89%
Hemorrhagic stroke	4	0.80%
Both types of stroke	363	72.31%
**Stage of dysphagia (*****n*** = **52)**		
Oral stage	9	17.31%
Pharyngeal phase	20	38.46%
Esophageal phase	1	1.92%
Oral stage and Pharyngeal phase	18	34.62%
Both stages of dysphagia	4	7.69%

#### 3.4.1 Participants

One hundred seventy-six studies involved more than 100 participants, while the remaining 639 studies involved 100 or fewer participants. Thirty-six studies reported the calculation method of sample size (33 for RCTs, 1 for nRCTs, and 2 for cohort studies) 0.505 studies reported the mean age of the participants, with 39.21% within 60-65 years old. 511 studies reported the mean duration of PSD. 502 studies reported the types of stroke, 52 studies reported the stages of dysphagia.

#### 3.4.2 Diagnostic criteria

The diagnostic criteria for the included studies were divided into stroke diagnostic criteria and dysphagia diagnostic criteria. The most common method used to diagnose stroke was transcranial computed tomography (CT) or magnetic resonance imaging (MRI) (*n* = 268, 32.88%), and six sets of diagnostic criteria were used more than 40 times in the studies, namely: “Diagnostic Points of Major Cerebrovascular Diseases in China” (*n* = 248, 30.43%), “Criteria for Diagnosis and Efficacy Evaluation of Stroke (trial)” (*n* = 97, 11.90%), “Guidelines for Cerebrovascular Disease Prevention and Treatment in China” (*n* = 57, 6.99%), “Chinese Guidelines for Diagnosis and Treatment of Intracerebral Hemorrhage”(*n* = 46, 5.64%), “Chinese Guidelines for Diagnosis and Treatment of Acute Ischemic Stroke” (*n* = 45, 5.52%) and “Diagnostic Criteria of Stroke Formulated in the Fourth National Conference on Cerebrovascular Diseases in 1995” (*n* = 43, 5.28%).

The diagnosis of dysphagia involved four diagnostic criteria that were used more than 40 times in the studies, as follows: “Kubota Water Swallowing Test (WST)” (*n* = 251, 30.80%), “Video fluoroscopic swallowing study (VFSS)” (*n* = 72, 8.83%), <Practical Neurology> (*n* = 52, 6.38%), and <Neurology> (*n* = 46, 5.64%).

#### 3.4.3 Interventions

A total of 488 studies reported the period of intervention and 48 studies reported the follow-up period. 754 studies reported the duration of intervention, <4 weeks of intervention was used as frequently as 659 times is nearly 87.40%. 707 studies reported the times of acupuncture treatment, <20 times were used as frequently as 407 times, which was more than half of all frequencies. This may indicate that the PSD recovered better for a shorter duration with acupuncture interventions (Table 2). The interventions in the studies included manual acupuncture (MA) (*n* = 103, 12.64%), electric acupuncture (EA) (*n* = 39, 4.79%), MA combined western medicine therapy (*n* = 441, 54.11%), EA combined western medicine therapy (*n* = 159, 19.51%), MA/EA combined TCM therapy (*n* = 18, 2.21%), MA/EA combined Chinese and western medicine therapy (*n* = 55, 6.75%). The control groups were no-treatment (*n* = 22, 2.79%), western medicine therapy (*n* = 439, 55.64%), integrated traditional Chinese and Western medicine therapy (*n* = 18, 2.28%), acupuncture (*n* = 65, 8.24%), acupuncture combined with TCM and/or western medicine therapy (*n* = 214, 27.12%), and sham acupuncture (*n* = 3, 0.38 %).

**Table 2:** Intervention characteristics of the included clinical studies.

**Intervention characteristics**	**Studies no**.	**(%)**
**Timing of intervention (*****n*** = **488)**		
Acute phase	70	14.34%
Remission phase	242	49.59%
Sequelae phase	19	3.89%
Acute phase and Remission phase	132	27.05%
Remission phase and Sequelae phase	8	1.64%
Acute phase, Remission phase and Sequelae phase	17	3.48%
**Period(*****n*** = **754)**		
≤ 4 weeks	659	87.40%
4–8 weeks	85	11.27%
>8 weeks	10	1.33%
**Number of acupuncture treatment(*****n*** = **707)**		
≤ 10	158	22.35%
10–20	249	35.22%
21–30	232	32.81%
>30	68	9.62%
**Follow-up period(*****n*** = **48)**		
1 month	14	29.17%
2 month	5	10.42%
3 month	18	37.50%
>3 month	11	22.92%

#### 3.4.4 Acupuncture treatment

A total of 1,108 acupuncture prescriptions were reported in 815 clinical original studies, and the characteristics are shown in Tables 3, 4. There were 168 acupoints involved in the study, and Lianquan (RN23) was the most commonly used acupoint. The different methods used during the needling manipulation produced different amounts of stimulation. A total of 794 acupuncture prescriptions recorded the dose–effect, such as direction, intensity, depth, and manipulation. 586 (52.89%) acupuncture prescriptions selected the tonifying and/or relieving diarrhea method. A total of 971 acupuncture prescriptions recorded retention time, 30 min was the most commonly used retention time (694, 71.47%).

**Table 3:** Distribution of acupuncture protocols.

**Characteristics**	**Number**	**(%)**
**Acupuncture prescriptions (*****n*** = **1,108)**		
Single acupoint	35	3.16%
Multiple acupoints	1,073	96.84%
**Acupoints compatibility (*****n*** = **1,108)**		
local point selection	413	37.27%
Distal point selection	50	4.51%
Distal-proximal point association	645	58.21%
**Number of needles (*****n*** = **347)**		
1 ≤ n ≤ 5	118	34.01%
5 <n ≤ 10	112	32.28%
10 <n ≤ 15	76	21.90%
*n* > 15	41	11.82%
**Acupuncture protocols (*****n*** = **1,108)**		
MA	471	42.51%
EA	255	23.01%
MA+Blood-letting	268	24.19%
EA+Blood-letting	58	5.23%
MA+acupuncture-related methods	49	4.42%
EA+acupuncture-related methods	7	0.63%
**Expert experience program (*****n*** = **110)**		
Xingnao Kaiqiao acupuncture therapy	37	33.64%
Tri-tongue acupuncture therapy	25	22.73%
Tongguan Liqiao acupuncture therapy	16	14.55%
Jin's three-needle acupuncture therapy	10	9.09%
Canggui Tanxue Technique	7	6.36%
Tongdutiaoshen acupuncture therapy	6	5.45%
Yu's scalp acupuncture therapy	5	4.55%
Penetrating-needling and swallowing technique of acupuncture	4	3.64%

**Table 4:** Distribution of acupuncture sites.

**Characteristics**	**Number**	**(%)**
^*^Acupuncture sites (***n*** = **1,108)**		
Body acupuncture	467	42.15%
Tongue acupuncture	384	34.66%
Scalp acupuncture	194	17.51%
Nuchal acupuncture	29	2.62%
Eye acupuncture	16	1.44%
Auricular acupuncture	10	0.90%
Abdominal acupuncture	6	0.54%
Neck acupuncture	2	0.18%
**Selected acupoints (*****n*** = **1,108)**		
Lianquan (RN23)	686	61.91%
Fengchi (GB20)	653	58.94%
Yifeng (SJ17)	344	31.05%
Yuye (EX-HN13)	329	29.69%
Jinjin (EX-HN12)	298	26.90%
Wangu (GB12)	244	22.02%
Neiguan (PC6)	198	17.87%
Fengfu (DU16)	167	15.07%
Sanyinjiao (SP6)	161	14.53%
Hegu (LI4)	150	13.54%

^*^A single acupuncture prescription may include 2 or more acupuncture sites.

### 3.5 Outcomes

**Figure 3:**
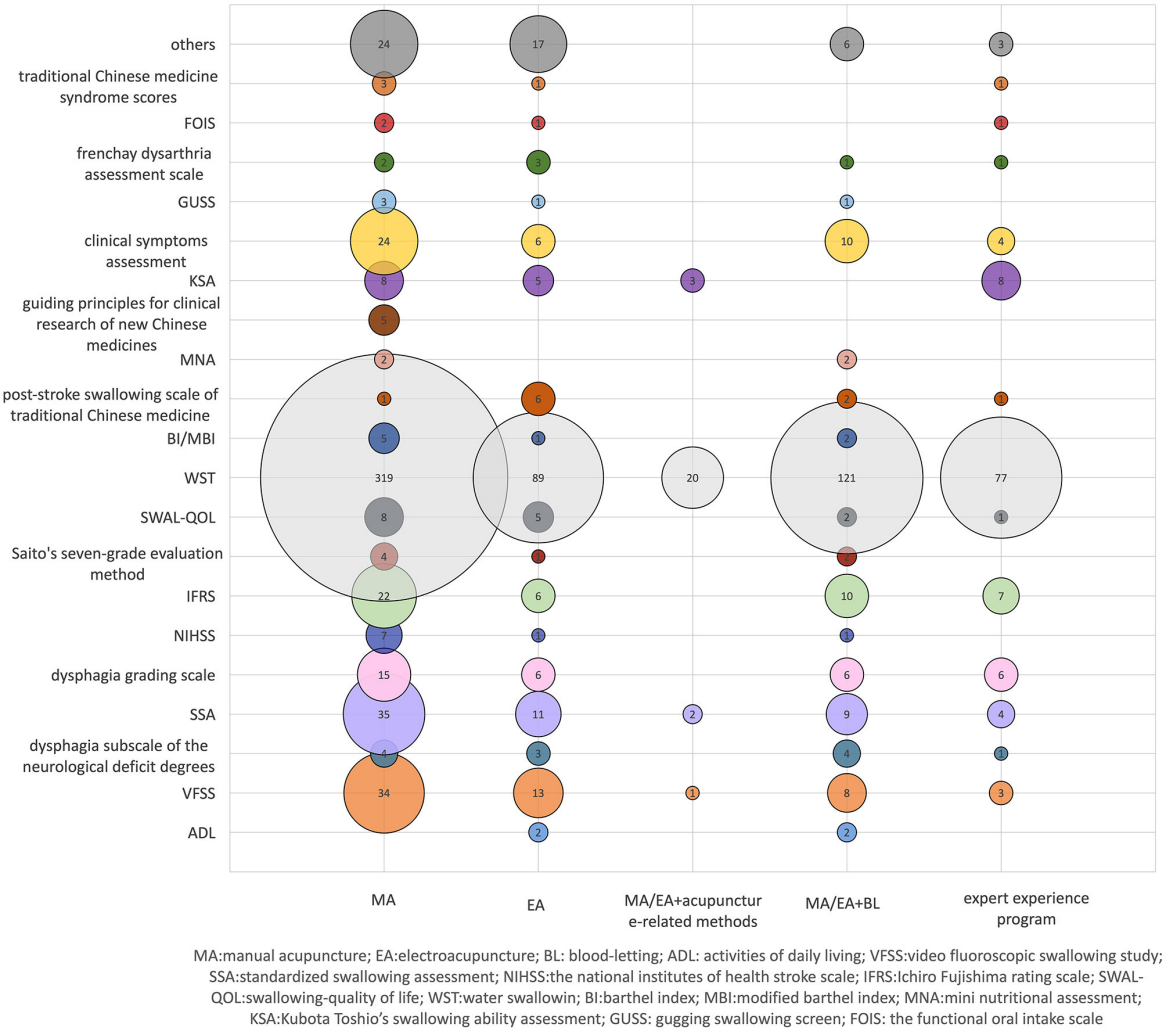
Effective evaluation criteria and reference source distribution. X-axis indicates different intervention methods, and the Y-axis represents the evaluation criteria and reference sources of effectiveness. The bubble size and the labeled values show the criteria and the frequency of use of reference sources.

A total of 815 clinical studies were analyzed, involving 119 outcome indicators. According to their characteristics, they could be divided into 12 categories, including swallowing function evaluation, mental psychology, behavioral characteristics, activities of daily living, TCM symptoms and signs, nutritional assessment, imaging examination, blood tests, language function, neurological and motor function, adverse reactions and complications, effective rate, others (including medical costs, compliance, satisfaction score of treatment, etc.). The most frequently used outcome index was effective rate (*n* = 668,81.96%), followed by water-swallowing test (WST) (*n* = 564,69.20%) (Table 5). In addition to commonly used rating scales and imaging tests, two studies used inflammatory factors interleukin (IL)-6 as evaluation criteria. 26 studies used neurocytokines and monoamine neurotransmitters as evaluation criteria, including brain-derived neurotrophic factor (BDNF), nerve growth factor (NGF), 5-HT, norepinephrine (NE), neuron-specific enolase (NSE), and vascular endothelial growth factor (VEGF). The evaluation criteria of efficiency and their reference sources are not uniform, and the most frequently used is WST (93.71%) (Figure 3).

**Table 5:** Outcome indicators.

**Outcome indicator**	**Number**	**(%)**
Effective Rate (ER)	668	81.96%
Kubota Water Swallowing Test (WST)	564	69.20%
Standardized Swallowing Assessment (SSA)	213	26.13%
Video fluoroscopic swallowing study (VFSS)	172	21.10%
Adverse reactions and complications	114	13.99%
Swallowing-quality of life (SWAL-QOL)	110	13.50%
Ichiro Fujishima rating scale (IFRS)	98	12.02%
National Institutes of Health Stroke Scale (NIHSS)	41	5.03%
Modified Barthel Index (MBI)	30	3.68%
Dysphagia grading scale	27	3.31%
sEMG	25	3.07%
Post-stroke swallowing scale of traditional Chinese medicine	24	2.94%
Functional Oral Intake Scale (FOIS)	24	2.94%
Activities of Daily Living (ADL)	23	2.82%
Hemorheological parameters	23	2.82%
Kubota Toshio's swallowing ability assessment	19	2.33%
Modified Mann assessment of swallowing ability (MMASA)	16	1.96%
Barthel Index (BI)	15	1.84%
Dysphagia subscale of the neurological deficit degrees	14	1.72%
Saito's seven-grade evaluation method	14	1.72%
Repetitive Saliva Swallowing Test (RSST)	14	1.72%

### 3.6 Adverse events

**Figure 4:**
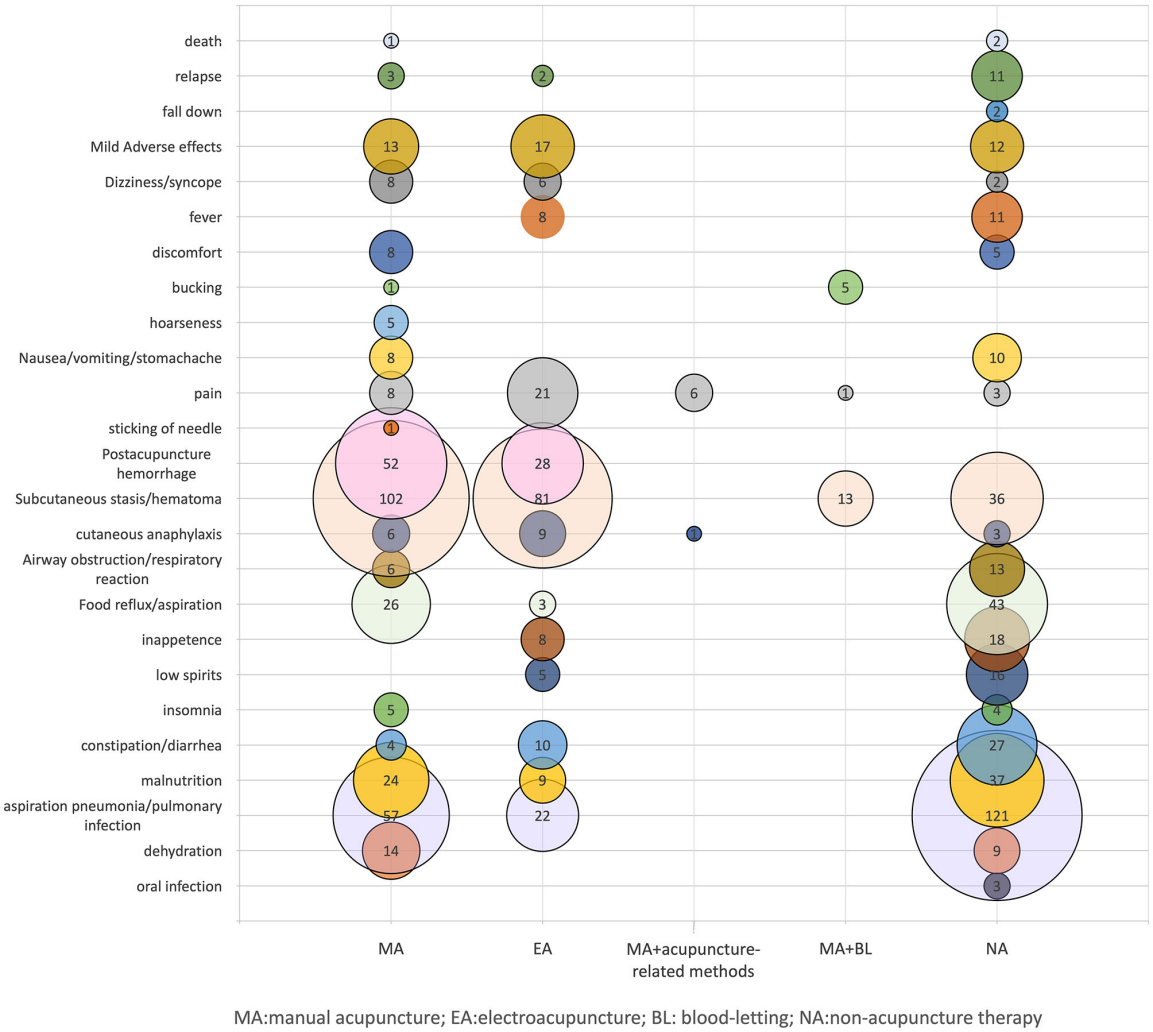
Distribution of adverse events and complications. X-axis indicates different intervention methods, Y-axis represents adverse reactions and complications. The bubble size and the labeled values show the number of adverse events and complications.

Adverse events and complications were reported in 114 studies. No adverse events were observed in 16 of these studies. There were 599 cases of adverse reactions reported by acupuncture interventions, including 381 cases of adverse reactions related to intervention measures, including skin discomfort, subcutaneous stasis, pinhole bleeding after acupuncture, etc., all of which were mild and relieved by themselves. Other complications occurred in 218 cases, including dehydration, aspiration pneumonia/pulmonary infection, malnutrition, constipation/diarrhea, food reflux/aspiration, airway obstruction/respiratory reaction, etc. There were 388 cases of adverse reactions reported by non-acupuncture interventions, including 61 cases of adverse reactions related to interventions and 327 cases of other complications (Figure 4).

### 3.7 Characteristics of included systematic reviews

As for the types of studies included, 43 SRs included only RCTs, 8 SRS included RCTs and qRCTs. The main studies included in each SR ranged from 1 to 72, and the number of subjects ranged from 66 to 6,134. Regarding the type of intervention, 4 SRs (7.84%) included acupuncture alone, 24 SRs (47.06%) included acupuncture combined with other therapies (including rehabilitation or swallowing training, medication, baseline treatment, etc.), and 23 SRs (45.10%) included acupuncture and acupuncture combined with other therapies (including rehabilitation or swallowing training, medication, baseline treatment, etc.). The primary outcome was heterogeneous and was defined differently in all included SRs. Efficiency (ER) and water swallow tests (WST) were frequently used. Video fluoroscopic swallowing study (VFSS), standardized swallowing assessment (SSA), Ichiro Fujishima rating scale (IFRS), incidence of adverse events and complications were also used as outcome measures.

All studies used quality assessment tools, the most commonly used was the Cochrane risk of bias tool (43, 84.31%). A total of 23 SRs (45.10%) mentioned adverse events and 19 SRs (37.25%) reported adverse events related to acupuncture, including pain at the acupuncture site, ecchymosis, hematoma, etc. No serious side effects were reported. These data proved the safety of acupuncture in the treatment of dysphagia after stroke. 26 SRs (50.98%) showed that the quality of evidence on the effectiveness of acupuncture in the treatment of dysphagia after stroke was low, and high-quality trials with large sample sizes were still needed. Supplementary Table S2 lists the characteristics of these included SRs.

### 3.8 Guidance

Acupuncture provided positive recommendations in nine guidelines for the treatment of PSD, and there was 1 negative statement regarding acupuncture use (Table 6).

**Table 6:** Treatment Guidelines (TG) that have made recommendations about the use of acupuncture.

**Clinical practice guidelines**	**Year**	**Country**	**Recommendation**
National clinical guideline for stroke 5th edition (Royal College of Physicians, 2016)	2016	UK	**(+)** There was some evidence that acupuncture … may reduce dysphagia
Palliative and end-of-life care in stroke: a statement for healthcare professionals from the American Heart Association/American Stroke Association (Holloway et al., 2014)	2014	US	**(+)** Acupuncture may reduce the proportion of patients with persistent dysphagia
Guidelines for Adult Stroke Rehabilitation and Recovery. A Guideline for Healthcare Professionals from the American Heart Association/American Stroke Association (Winstein et al., 2016)	2016	US	**(+)** Acupuncture may be a beneficial alternative treatment of dysphagia (**Class-IIb; Level of evidence:B**).
Canadian Best Practice Recommendations for Stroke Care. Fourth Edition (Canadian Best Practice Recommendations for Stroke Care, 2013)	2013	Canada	(+) Acupuncture and behavioral modifications were associated with reduction in the presence of dysphagia at the end of treatment.
Evidence based review of stroke rehabilitation (18th edition) (Canadian Partnership for Stroke Recovery, 2018)	2018	Canada	(+) Acupuncture combined with physical therapy can be an effective treatment for dysphagia, however more research is required to strengthen this treatment protocol (**Level of evidence: II**).
Guideline for the diagnosis and treatment of post-stroke dysphagia (Dziewas et al., 2021)	2021	Europe	(+) Recommends in patients with post-stroke dysphagia, acupuncture may be used to rehabilitate swallowing function (**Moderate Strength;Weak for intervention↑**).
Clinical practice guidelines for traditional Chinese medicine rehabilitation of stroke (Lin et al., 2019)	2019	China	(+) Acupuncture combined with swallowing rehabilitation is beneficial to the recovery of swallowing dysfunction after stroke (**Level of evidence:B**).
Clinical practice guidelines for traditional Chinese medicine rehabilitation of ischemic stroke (cerebral infarction) (Zhang et al., 2021)	2021	China	(+) Acupuncture can help improve the swallowing function of patients with ischemic stroke in the recovery stage and the sequelae stage (**Grading of recommendation: II, Level of evidence:C**).
Evidence-based Chinese Medicine Clinical Practice Guideline for Stroke in Hong Kong (Zhong et al., 2020)	2020	China	(+) There is evidence demonstrating the effectiveness of acupuncture in the treating dysphagia, but a higher level of evidence-based studies is still required (**Grading of recommendation: B, Level of evidence: IIa**).
2017 Clinical Guidelines (Stroke Foundation, 2017)	2017	Australia	(–) For patients with stroke, acupuncture should not be used for treatment of dysphagia in routine practice other than as part of a research study (**Weak recommendation**).

(+) Recommendation that acupuncture should be used; (–) Recommendation that acupuncture should not be used.

## 4 Discussion

Post-stroke dysphagia is in the category of “unsound speech and motor impairment” in TCM. It is believed in TCM that the etiology and pathogenesis of dysphagia are pathogenic wind, fire, phlegm, blood stasis, and qi deficiency. Acupuncture can be used at the corresponding points to nourish yin, activate collaterals, wake up the brain, open the aperture, and remove obstructions (Zhong et al., 2021). Some studies (Zheng et al., 2023; Zhang et al., 2024) showed that acupuncture therapy could effectively improve brain circulation and energy metabolism, promote neural remodeling of infarction region, increase the corticobulbar tract volume, activate the specific motor functional areas of the cerebral cortex, accelerate the recovery of central nervous system function, coordinate the movement of pharyngeal muscles, and promote the rehabilitation of swallowing function. This may be the main mechanism of acupuncture in treating PSD. Our scoping review examined the breadth and nature of acupuncture use in the treatment of PSD, identified 815 original clinical studies, 51 systematic reviews, 254 reviews, and 10 guidelines exploring the efficacy and safety of acupuncture. Most reviews and studies reported positive results affirming the efficacy of acupuncture for PSD.

### 4.1 Intervention time of acupuncture

Acupuncture could improve swallowing at different stages of treatment. The timing of acupuncture intervention, duration, and the number of acupuncture treatments received varied widely among the studies. There is no conclusion on the relationship between the timing of acupuncture intervention and the curative effect. It has been suggested that acupuncture should ideally be used in the first 3 months after a stroke and as soon as the patient is stable and receiving medication and rehabilitation to enhance or accelerate recovery after stroke (Xu and Fan, 2012). Some studies (Xia et al., 2016; Long, 2023) have shown that acupuncture intervention and rehabilitation therapy in the acute phase can better improve the swallowing function of patients and improve the curative effect. The earlier and longer the duration of acupuncture intervention, the better the recovery of swallowing function, which may be related to the time required to remodel brain function (Gao and Zhou, 2020). However, a systematic review found that the subgroup with a disease duration of more than 3 years had better VFSS scores than the <6 months' subgroup (Jiang et al., 2022). Therefore, the timing of acupuncture intervention in the acute stage and recovery stage of stroke needs to be further clarified in future studies.

### 4.2 Acupuncture protocol

Among the included articles, acupuncture treatment is usually combined with other traditional Chinese medicine (herbal medicine) or oral Western medicine and rehabilitation training. According to acupoint compatibility, the acupuncture schemes for PSD can be divided into two categories: Local acupuncture therapy, mainly tongue acupuncture and scalp acupuncture; Comprehensive acupuncture therapy based on the Xingnao Kaiqiao acupuncture method. These acupuncture methods can all have a positive effect on the rehabilitation of swallowing function, and there is complementarity in acupoint selection and therapeutic effect among different acupuncture methods. The comprehensive acupuncture program combining the advantages of different acupuncture methods may have more benefits and be more in line with the holistic concept of traditional Chinese medicine. The commonly used acupoints for post-stroke dysphagia summarized in this review are mainly distributed in the head, face, and neck, which is consistent with the etiology of dysphagia and the location of lesions (cerebral cortex or subcortex). Therefore, acupuncture at the head acupoints stimulates the corresponding swallowing function area of the brain, improves cerebral blood flow, regulates growth factors, reduces inflammatory factors, and promotes the recovery of neuronal cells (Wu et al., 2024). The commonly used acupoints reflect the treatment principle of “where the acupoints are, where the indications are”. Among them, Lianquan (RN23) is an important acupoint mainly for deglutition disorder, and it can be used to benefit the pharynx (Xia et al., 2016). Fengchi (GB20) can be used to suppress yang, extinguish wind, and dissolve phlegm (Liu et al., 2012). Yifeng (SJ17) can be used to open depression winds and benefit the pharynges (Feng et al., 2016). Jinjin (EX-HN12) and Yuye (EX-HN13) are acupoints for dredging meridians, regulating and smoothening qi and blood (Gao and Zhou, 2020). These acupoints could open the aperture, wake up the brain, activate the collaterals, remove obstructions, benefit pharynges, and promote the recovery of patients with PSD. In addition, LR3 and SP6 can be added for patients with hyperactivity of liver Yang; ST40 and ST36 can be applied for patients with wind-phlegm blocking collaterals; ST40, SJ6 and LI11 can be applied for patients with phlegm-heat and bowel repletion; SP10, ST36 and RN6 can be added for patients with qi deficiency and blood stasis; KI3 and SP6 can be added for patients with wind formation from yin deficiency; KI3, KI7, LR3 and SP6 can be applied for patients with liver-kidney yin deficiency.

In the physiological state, swallowing activity is divided into the preoral phase, oral preparation phase, oral phase, pharyngeal phase, and esophageal phase. Due to the different locations of motor neurons damaged after a stroke, patients exhibit functional impairment in different swallowing periods, and the local locations of the disease are different. The patient's lesion location, stroke severity, and etiology can significantly impact the prognosis of these patients (Sun et al., 2023). Acupuncture intervention should refine the pathological stages and lesions of swallowing and select acupuncture prescriptions specifically. There are many acupoints selection schemes in previous studies, and there is no unified standard. The selection of acupoints should not only treat the primary lesion but also consider the local functional recovery. It is necessary to conduct research on the specificity of meridian acupoints under different pathological states of swallowing, screen meridian acupoints with specific therapeutic effects, optimize the clinical acupoint selection scheme for acupuncture therapy, and improve the clinical efficacy of acupuncture.

In addition to the acupoint selection scheme, the efficacy of acupuncture is also related to the acupuncture treatment dose, including the total number and frequency of treatments. A systematic review found that the subgroup with more than 20 treatments had better WST scores than the subgroup with fewer than 20 treatments (Jiang et al., 2022). A meta-analysis has found that, compared with acupuncture treatment course ≤ 30 days group, the >30 days group result revealed a higher effect size than the control conditions (Luo et al., 2023). Due to the limited stimulation by a single acupuncture treatment, it is necessary to continue treatment for a period of time to accumulate effects. However, acupuncture tolerance may also result from long-term and repeated acupuncture stimulation, which has negative effects or even leads to more adverse events (Wang B. et al., 2023). More studies are needed to clarify the dose-effect relationship of acupuncture.

### 4.3 Clinical outcome indicator

Among the outcome indicators, the most frequently used tool to measure the effectiveness of acupuncture on dysphagia after stroke was the efficacy rate (ER). However, the concept of ER in these studies varies, most studies did not provide a clear definition of ER and had various reference standards, makes it difficult to summarize and compare data between different studies. The clinical efficacy evaluation of acupuncture in the treatment of PSD mainly involves swallowing function, neurological function, TCM syndrome, quality of life, mental state, and nutritional status. Several SRs have reported heterogeneity in outcome reporting in many clinical trials (Zhong et al., 2021; Tang et al., 2022), inconsistent outcome reporting creates barriers to data synthesis and comparison, leading to a waste of study resources. Therefore, it is necessary to combine the characteristics of acupuncture treatment with the current status of clinical research and develop a core outcome set (COS) with TCM characteristics to improve the quality of outcome reporting. Our scoping review generated a list of potentially important outcomes for PSD COS development. Most studies focus on swallowing function scores, imaging examinations, and neurological function evaluations. However, less attention was paid to psychology, TCM symptoms and signs, nutritional status, and blood tests. The assessment tools include simple scales, instruments, laboratory indicators, etc. One study found that the most frequently reported measurement tools in randomized controlled trials of acupuncture for post-stroke dysphagia included WST, IFRS, SSA, VFSS, and SWAL-QOL (Cao et al., 2023), which were consistent with the results of our scoping review. Clinical studies of post-stroke dysphagia were mostly observed using subjective indicators. WST is the most classical and concise screening method for dysphagia, while SSA is an internationally recognized tool for the assessment of dysphagia with the advantages of easy accessibility and patient tolerance. Some studies have analyzed the reliability and validity of these commonly used dysphagia measurement tools. WST has good reliability but low validity, which can be affected by patients' subjective feelings, potentially making aspiration screening results unreliable. Both SSA and IFRS have good reliability and validity. IFRS can predict pneumonia during hospitalization and assess nutritional status at discharge. SWAL-QOL is a specific scale designed to assess the quality of life in patients with dysphagia, which has good reliability and validity (Niu et al., 2020; Lai et al., 2021). However, compared with instrumental examination, the evaluation results of these scales lack objectivity and make it difficult to quantitatively analyze various aspects of the patient. Some studies (Zhuang et al., 2022)have shown that acupuncture can enhance the activity of brain areas related to swallowing and promote the recovery of swallowing function in patients with dysphagia. The efficacy of acupuncture can be observed in combination with brain imaging techniques such as functional magnetic resonance imaging (fMRI), functional near-infrared spectroscopy (fNIRS), electroencephalogram (EEG), magnetoencephalogram (MEG), diffusion tensor imaging (DTI), and arterial spin labeling (ASL). A multi-modal design is helpful to comprehensively explain the mechanism of acupuncture treatment (Hernandez et al., 2019; Bhutada et al., 2022; Fu et al., 2023; Qin et al., 2023; Zhang Z. et al., 2023; Zheng et al., 2023).

In addition to the outcome indicators related to the treatment, the evaluation of clinical efficacy also includes the health resources invested in the intervention. The cost of stroke treatment and hospitalization imposes a heavy economic burden on medical systems around the world (Rajsic et al., 2019). How to allocate and use the limited health resources reasonably and control and reduce unnecessary cost consumption has become a subject of increasing interest among researchers. A real-world study (Zhang, 2020) included in this review evaluated acupuncture therapy from the perspective of health economics and found that the direct medical costs and direct non-medical costs of acupuncture and rehabilitation therapy were lower than those of the rehabilitation group. Additionally, the cost-effectiveness ratio of acupuncture and rehabilitation therapy was much smaller than that of the rehabilitation group. A retrospective study (Zhao, 2020) found that early acupuncture was an independent risk factor for discharge time. This suggests that early acupuncture intervention may shorten the hospitalization time of patients with dysphagia after stroke, reduce the economic burden, and offer a cost-effectiveness ratio advantage. However, the existing clinical evidence cannot fully reflect the therapeutic value of acupuncture, which complicates the health economic evaluation of acupuncture interventions and is not conducive to the formulation of relevant policies. More studies are needed to provide a basis for medical and health decision-making from the perspective of health economics.

The modern clinical mode of simultaneous physical and psychological treatment is the commonly applied general trend, as participant beliefs and experiences in acupuncture and moxibustion are also part of the curative effect. Qualitative data from this aspect is currently missing and required (Enck et al., 2010; Liu et al., 2022). Relevant information about the participants' acupuncture treatment experience can be collected through interviews and acupuncture sensation assessment scales, to enrich the dimensions of the interpretation of research results.

### 4.4 Assessment of clinical evidence

Acupuncture is safe and effective in the treatment of post-stroke dysphagia (Li et al., 2019; Tian et al., 2019), which has been recommended by clinical guidelines, but the strength of recommendation and the level of evidence are not high, indicating that acupuncture is not widely promoted in the world. Only a few healthcare systems have incorporated acupuncture into clinical practice guidelines and national health coverage for these conditions (Thomas and Coleman, 2004; Xue et al., 2008; Bleck et al., 2021), so both physicians and patients do not choose acupuncture as a treatment, and guideline makers do not include acupuncture in their recommendations. On the other hand, the evidence chain of acupuncture and moxibustion in the treatment of post-stroke dysphagia is not complete, and many studies on acupuncture and moxibustion intervention therapy have not been conducted in accordance with international research standards. Most of the articles on acupuncture and moxibustion in the treatment of post-stroke dysphagia are in Chinese, which hinders the promotion of research abroad, and the quality of evidence in some studies is poor. Therefore, most guidelines do not include acupuncture as a recommendation. Existing studies have found that evidence used to support recommendations often fails to report key information, target populations, and lacks sufficient details about acupuncture interventions, patient outcomes, and other factors (Birch and Robinson, 2022). If the evidence is not conclusive, the implementation of recommendations remains problematic, and it is emphasized that relevant reporting standards should be followed.

### 4.5 Limitations

There are several limitations to this study. Due to the language restriction, we only included articles published in English and Chinese, and most of the included studies were reported in China, which may lead to the omission of eligible research. Moreover, the intention of a scoping review is simply to summarize the breadth of the available literature, and it refrains from assessing the quality and publication bias of the included studies. There is obvious heterogeneity in the included articles and this review cannot provide answers to more specific questions like a systemic review does. Due to the large number of studies included, there may be oversimplification of the manipulation of acupuncture methods and findings in the results. More detailed information on study design and intervention parameters (including randomization method, needle type, electroacupuncture waveform, frequency, current intensity, etc.) should be collected according to different research purposes in future studies.

### 4.6 Conclusion

This review systematically summarized the research status of acupuncture therapy in the treatment of PSD, including diagnostic criteria, acupuncture protocols, outcome indicators, adverse events, and clinical evidence. As a convenient and safe traditional Chinese medicine therapy with its own characteristics, acupuncture can improve different stages and types of dysphagia without serious adverse reactions. In the future, more standardized international cooperative clinical research is needed to identify the influence of different acupuncture intervention times on the curative effect and dose-effect relationship of acupuncture; standardize the clinical acupoint selection scheme of acupuncture; develop a COS with TCM characteristics to improve the quality of outcome reporting and make different research data can be summarized and compared, reduce the waste of medical resources, and provide more high-quality evidence.
