# Supplementary material for: Current state of research on acupuncture for the treatment of post-stroke dysphagia: a scoping review
Source: Front Neurosci. 2024 Aug 15;18:1391576. doi: 10.3389/fnins.2024.1391576 (PMC11357938; doi:10.3389/fnins.2024.1391576)
Supplement: Supplementary file 1 [file Table_1.docx]

Supplementary Material

# Table S1: Search strategies for each database

CNKI Search:

((SU='针刺' or TI='针刺' or KY='针刺' or AB='针刺' or SU='针灸' or TI='针灸' or KY='针灸' or AB='针灸' or SU='电针' or TI='电针' or KY='电针' or AB='电针' or SU='针' or TI='针' or KY='针' or AB='针') and (SU='脑梗死' or TI='脑梗死' or KY='脑梗死' or AB='脑梗死' or SU='脑出血' or TI='脑出血' or KY='脑出血' or AB='脑出血' or SU='脑栓塞' or TI='脑栓塞' or KY='脑栓塞' or AB='脑栓塞' or SU='卒中' or TI='卒中' or KY='卒中' or AB='卒中' or SU='中风' or TI='中风' or KY='中风' or AB='中风') and (SU='吞咽困难' or TI='吞咽困难' or KY='吞咽困难' or AB='吞咽困难' or SU='吞咽障碍' or TI='吞咽障碍' or KY='吞咽障碍' or AB='吞咽障碍' or SU='假性延髓麻痹' or TI='假性延髓麻痹' or KY='假性延髓麻痹' or AB='假性延髓麻痹' or SU='假性球麻痹' or TI='假性球麻痹' or KY='假性球麻痹' or AB='假性球麻痹')).

VIP Search:

((M=针刺 OR 针灸 OR 电针 OR 针) OR R=针刺 OR 针灸 OR 电针 OR 针) AND (M=脑梗死 OR 脑出血 OR 脑栓塞 OR 卒中 OR 中风) OR R=脑梗死 OR 脑出血 OR 脑栓塞 OR 卒中 OR 中风) AND (M=吞咽困难 OR 吞咽障碍 OR 假性延髓麻痹 OR 假性球麻痹) OR R=吞咽困难 OR 吞咽障碍 OR 假性延髓麻痹 OR 假性球麻痹)

Wanfang Search:

主题:("针刺" OR "针灸" OR "电针" OR "针") and 主题:( "脑梗死" OR "脑出血" OR "脑栓塞" OR "卒中" OR "中风") and 主题:("吞咽困难" OR "吞咽障碍" OR "假性延髓麻痹" OR "假性球麻痹")

SinoMed Search:

(("针刺"[不加权:扩展] OR "电针"[不加权:扩展]) OR “针刺”[常用字段:智能] OR “针灸” [常用字段:智能] OR “电针” [常用字段:智能] OR “针” [常用字段:智能])) AND (("中风"[不加权:扩展]) OR “中风” [常用字段:智能] OR “脑梗死” [常用字段:智能] OR “脑出血” [常用字段:智能] OR “脑栓塞” [常用字段:智能] OR “卒中” [常用字段:智能])) AND (("吞咽障碍"[不加权:扩展]) OR “吞咽困难” [常用字段:智能] OR “吞咽障碍” [常用字段:智能] OR “假性延髓麻痹” [常用字段:智能] OR “假性球麻痹” [常用字段:智能]))

Embase Search:

#4 #1 AND #2 AND #3

#3 'dysphagia'/exp OR dysphagia:ti,ab,kw OR 'pseudobulbar palsy'/exp OR 'pseudobulbar palsy':ti,ab,kw OR 'swallowing disorders' OR 'swallowing disorders':ti,ab,kw OR 'deglutition disorders'/exp OR 'deglutition disorders':ti,ab,kw

#2 'stroke'/exp OR stroke:ti,ab,kw OR 'stroke cerebral' OR 'stroke cerebral':ti,ab,kw OR 'brain infarction'/exp OR 'brain infarction':ti,ab,kw OR 'cerebral infarction'/exp OR 'cerebral infarction':ti,ab,kw OR 'intracranial hemorrhage'/exp OR 'intracranial hemorrhage':ti,ab,kw OR 'cerebrovascular accident'/exp OR 'cerebrovascular accident':ti,ab,kw OR 'cerebral hemorrhage'/exp OR 'cerebral hemorrhage':ti,ab,kw

#1 'acupuncture'/exp OR 'electroacupuncture'/exp OR acupuncture:ti,ab,kw OR electroacupuncture:ti,ab,kw

Pubmed Search:

(((((acupuncture[MeSH Terms]) OR (acupuncture[Title/Abstract])) OR (electroacupuncture[MeSH Terms])) OR (electroacupuncture[Title/Abstract])) AND ((((((((stroke[MeSH Terms]) OR (stroke[Title/Abstract])) OR ("stroke cerebral"[Title/Abstract])) OR ("brain infarction"[Title/Abstract])) OR ("cerebral infarction"[Title/Abstract])) OR ("intracranial hemorrhage"[Title/Abstract])) OR ("cerebrovascular accident"[Title/Abstract])) OR ("cerebral hemorrhage"[Title/Abstract]))) AND ((((("deglutition disorders"[MeSH Terms]) OR (deglutition disorders[Title/Abstract])) OR ("dysphagia"[Title/Abstract])) OR ("swallowing disorders"[Title/Abstract])) OR ("pseudobulbar palsy"[Title/Abstract]))

Web of science Search:

#4 #1 AND #2 AND#3

#3 ((((((((((TS=(deglutition disorders)) OR TI=(deglutition disorders)) OR AB=(deglutition disorders)) OR TS=(dysphagia)) OR TI=(dysphagia)) OR AB=(dysphagia)) OR TS=(swallowing disorders)) OR TI=(swallowing disorders)) OR AB=(swallowing disorders)) OR TS=(pseudobulbar palsy)) OR TI=(pseudobulbar palsy)) OR AB=(pseudobulbar palsy)

#2 ((((((((((((((((((((TS=(stroke)) OR TI=(stroke)) OR AB=(stroke)) OR TS=(stroke cerebral)) OR TI=(stroke cerebral)) OR AB=(stroke cerebral)) OR TS=(brain infarction)) OR TI=(brain infarction)) OR AB=(brain infarction)) OR TS=(cerebral infarction)) OR TI=(cerebral infarction)) OR AB=(cerebral infarction)) OR TS=(intracranial hemorrhage)) OR TI=(intracranial hemorrhage)) OR AB=(intracranial hemorrhage)) OR TS=(cerebrovascular accident)) OR TI=(cerebrovascular accident)) OR AB=(cerebrovascular accident)) OR TS=(cerebral hemorrhage)) OR TI=(cerebral hemorrhage)) OR AB=(cerebral hemorrhage)

#1 (((((TS=(acupuncture)) OR TS=(electroacupuncture)) OR TI=(acupuncture)) OR TI=(electroacupuncture)) OR AB=(acupuncture)) OR AB=(electroacupuncture)

Cochrance library Search:

#1 MeSH descriptor: [Acupuncture] explode all trees

#2 MeSH descriptor: [Electroacupuncture] explode all trees

#3 MeSH descriptor: [Stroke] explode all trees

#4 MeSH descriptor: [Deglutition Disorders] explode all trees

#5 (acupuncture):ti,ab,kw OR (electroacupuncture):ti,ab,kw

#6 (stroke):ti,ab,kw OR (stroke cerebral):ti,ab,kw OR (brain infarction):ti,ab,kw OR (cerebral infarction):ti,ab,kw OR (intracranial hemorrhage):ti,ab,kw

#7 (cerebrovascular accident):ti,ab,kw OR (cerebral hemorrhage):ti,ab,kw

#8 (dysphagia):ti,ab,kw OR (pseudobulbar palsy):ti,ab,kw OR (swallowing disorders):ti,ab,kw OR (deglutition disorders):ti,ab,kw

#9 #1 OR #2 OR #5

#10 #3 OR #6 OR #7

#11 #4 OR #8

#12 #9 AND #10 AND #11

# Table S2. Characteristics of systematic reviews

|  | SRs | No.of Primary Studies | No.of Patients | Study Types | Intervention group | Control group | Adverse Effects | Outcome(s) | Evidence Quality Evaluation Tool | Main Conclusions |
| --- | --- | --- | --- | --- | --- | --- | --- | --- | --- | --- |
| 1 | Wang L 2006 | 7 | 506 | RCT/q-RCT | 1,2 | 3 | Subcutaneous hemorrhage, Postacupuncture hemorrhage | ER, death, VFSS, IAP | Cochrane risk of bias tool | A reliable conclusion cannot be drawn from the present data because of the low methodological quality, especially because of the lack of data on long-term outcomes. A tendency that acupuncture can improve dysphagia after stroke in short--term with no adverse effect is demonstrated. Therefore, it is necessary to conduct more large-scale, multicentral RCTs with high quality in future. |
| 2 | Xie Y 2008 | 1 | 66 | RCT | 2 | 3 | No | Resolution of dysphagia (defined as recovery of normal feeding, which includes solid food and water, but does not include pureed food) | Jadad score | There is not enough evidence to make any conclusion about the therapeutic effect of acupuncture for dysphagia after acute stroke. High quality and large scale randomized controlled trials are needed. |
| 3 | Zhao X 2010 | 13 | 1250 | RCT/q-RCT | 2 | 3,4 | Mentioned | ER, WST, VFSS, RSST | GRADE, Cochrane risk of bias tool | Acupuncture combined with swallowing function training is a relatively safe therapeutic measure that has shown a trend toward effectiveness in improving swallowing function. However, due to the low quality of included RCTs, well designed RCTs with large-scale and high-quality are still required. |
| 4 | Yuan M 2011 | 13 | 962 | RCT/q-RCT | 2 | 3 | No | ER,RR | Jadad score | Acupuncture combined with rehabilitation training is beneficial to the recovery of swallowing function for patients with post stroke dysphagia. However, more well-designed RCTs are needed to support this conclusion. |
| 5 | Li S 2011 | 7 | 1187 | RCT | 2 | 3 | No | ER, RR, dysphagia score, anarthria score, Healing time | Jadad score | Acupuncture therapy for pseudobulbar palsy is effective, but more high-quality RCTs are required to support this conclusion. |
| 6 | Long Y 2012 | 72 | 6134 | RCT | 2 | 3 | No | ER | Jadad score | Acupuncture might be beneficial in the rehabilitation of patients with dysphagia caused by stroke, and the evidence justifies future high-quality studies. |
| 7 | Zhu Y 2012 | 7 | 701 | RCT/q-RCT | 1,2 | 3 | No | ER, RR, WST, SFA | Cochrane risk of bias tool, Jadad score | Acupuncture can effectively improve the efficacy of pseudo-bulbar paralysis after stroke, but more RCTs with high-quality and large-sample size are needed. |
| 8 | Chen J 2015 | 8 | 170 | RCT | 1 | 3、4 | No | ER, IAP, AE | Cochrane risk of bias tool | Xingnao Kaiqiao(brain awakening through opening orifices)acupuncture method combined with conventional therapy (swallowing function training, medical treatment) benefits swallowing function recovery on patients with dysphagia after stroke. The evaluation of the timing, treatment, and concurrent treatment of dysphagia after stroke still requires well designed RCTs with large-scale and high-quality. |
| 9 | Hu T 2015 | 17 | 1158 | RCT | 2 | 3 | No | ER, RR | Jadad score | Acupuncture with points on neck was an effective treatment for post stroke dysphagia but more high quality RCTs to support this conclusion is still needed. |
| 10 | Xiang Y 2015 | 17 | 1440 | RCT | 1,2 | 3 | Subcutaneous hemorrhage | ER, WST, FIRS, VFSS, SSA, IAP, RR, AE | GRADE, Cochrane risk of bias tool | Acupuncture combined with drugs is better than simple drugs assessed by VFSS scores. Acupuncture combined with drugs and rehabilitation training is better than drugs combined with rehabilitation assessed by SSA and VFSS scores. Acupuncture has a positive effect on improving the WST, SSA and VFSS score. However, it has not been proven that acupuncture combined with drugs and rehabilitation training can reduce the incidence of aspiration pneumonia. Acupuncture combined with rehabilitation is better than rehabilitation training alone, but acupuncture and rehabilitation training have the same effect on the treatment of patients with post stroke dysphagia. However, the long-term effect of acupuncture on post stroke dysphagia is better than rehabilitation training. |
| 11 | Yu C 2016 | 9 | 577 | RCT | 1,2 | 3 | No | ER, WST | Jadad score | Acupuncture was efficacious in treating post stroke dysphagia, but still high-quality and large-sample-size RCTs are required to support this conclusion. |
| 12 | Meng D 2016 | 10 | 898 | RCT | 1,2 | 3 | No | ER | Jadad score | Both acupuncture treatment and acupuncture combined with swallowing function rehabilitation training are more effective in treating post stroke dysphagia (compared to other therapies). |
| 13 | Liu H 2016 | 14 | 1155 | RCT | 1,2 | 3 | Subcutaneous hemorrhage at local point | ER, RR, WST, dysphagia subscale of the neurological deficit degrees, FDS, removal rate of nasal feeding tube, MNA, BI, MAMC, TSF, VFSS | GRADE, Cochrane risk of bias tool | Conventional treatment plus acupuncture was more effective for some outcomes than conventional treatment alone, but RCTs with higher quality in the future may produce new evidence. |
| 14 | Ye Q 2017 | 71 | 6010 | RCT | 1、2 | 3 | Mentioned | ER, WST, SSA, SFA, SWAL-QOL, ADL, CT7R | Cochrane risk of bias tool | Acupuncture for dysphagia after stroke has therapeutic efficacy. And the acupuncture is safe and reliable within a certain range. More strict evaluation standard and high-quality RCT design are necessary for further exploration on the acupuncture for treatment of dysphagia after stroke. |
| 15 | Zhang Y 2017 | 12 | 824 | RCT | 1、2 | 3 | No | ER, WST | Cochrane risk of bias tool | Electro acupuncture was an effective treatment for post stroke dysphagia but still need more high quality RCTs to support this conclusion. |
| 16 | Zhang S 2017 | 11 | 735 | RCT | 1、2 | 3、4 | No | ER, WST, VFSS | Jadad score | Electroacupuncture has shown significant efficacy in post-stroke dysphagia, but more high-quality literature is needed to support this conclusion. |
| 17 | Wang C 2017 | 32 | 2831 | RCT | 2 | 3 | No | ER | Jadad score | Acupuncture treatment for post stroke dysphagia shows better clinical efficacy. Multi-center and large-sample RCTs are still needed to support this conclusion. |
| 18 | Xu M 2017 | 11 | 907 | RCT | 1 | 3 | No | ER | Cochrane risk of bias tool | Acupuncture therapy had better effects on post stroke dysphagia. |
| 19 | Liao M 2017 | 42 | 3268 | RCT/q-RCT | 1,2 | 3,4 | No | ER, WST | Cochrane risk of bias tool | Jin’s three-needle alone or combined with other therapies can effectively improve the efficacy of pseudo-bulbar paralysis after stroke, but more RCTs with high-quality and large-sample size are needed. |
| 20 | Li L 2018 | 29 | 2190 | RCT | 1 | 3 | Mentioned | ER, WST, KSA, FDS, VFSS, CSA, AE | Cochrane risk of bias tool, CONSORT, STRICTA | Acupuncture is an effective and safe alternative therapy for treatment to post-stroke dysphagia, although the beneficial effect from acupuncture is possibly overvalued due to the low methodology quality of the included RCTs. More high-quality and large-scale research studies are needed. |
| 21 | Shi L 2018 | 12 | 1012 | RCT | 2 | 3 | No | ER, WST | Cochrane risk of bias tool | Acupuncture combined with rehabilitation training increased the efficacy and reduced the degree of dysphagia in post stroke patients, but still more high-quality RCTs needed to support this conclusion. |
| 22 | Yang J 2018 | 8 | 667 | RCT | 1,2 | 3,4 | No | ER | Cochrane risk of bias tool | Tongue three-needle therapy is obviously effective in the treatment of post-stroke pseudobulbar palsy.However, due to the low quality of included RCTs, well designed RCTs with large-scale and high-quality are still required. |
| 23 | Wang Y 2018 | 18 | 1680 | RCT | 2 | 3 | Subcutaneous hemorrhage, Postacupuncture hemorrhage, pain | ER, WST, VFSS | GRADE, Cochrane risk of bias tool | Acupuncture combined with rehabilitation training can significantly improve clinical outcomes; however, the low quality of the original literature limits the credibility of the conclusions, and there is a need to continuously explore rigorous scientific methodology and to emphasize high-quality, large-sample, multicenter RCTs in order to obtain high-level evidence-based medicine. |
| 24 | Chen Y 2018 | 4 | 425 | RCT/q-RCT | 2 | 3 | No | ER, WST | Cochrane risk of bias tool | Compared with the conventional rehabilitation treatment group, the acupuncture treatment group had better improvements for post stroke dysphagia according to the WST, but still requires high-quality and large-sample-size RCTs to support this conclusion. |
| 25 | Li L 2019 | 17 | 1479 | RCT | 2 | 3 | pain, ecchymosis and hematoma | ER, SFA, IA, QOL, AE | Cochrane risk of bias tool | Acupuncture combined with swallowing training can improve the ER, SFA and IAs of daily life in patients with post stroke dysphagia compared with swallowing training alone. However, further clinical trials with large sample sizes and standardized methodology are required to verify these results. |
| 26 | Wang H 2019 | 12 | 921 | RCT | 2 | 3 | No | ER, WST, SSA, FDS, VFSS, SWAL-QOL | Cochrane risk of bias tool | Acupuncture combined with rehabilitation training can effectively treat post-stroke dysphagia. Due to the low quality of the literature included in the study, more high-quality, large sample size, double-blind, multicenter randomized clinical trials are needed. |
| 27 | Xu L 2019 | 22 | 1998 | RCT | 1,2 | 3,4 | No | ER, RR, WST, FIRS, SSA | Cochrane risk of bias tool, Jadad score, GRADE | Xingnao Kaiqiao(brain awakening through opening orifices)acupuncture method is effective for post-stroke dysphagia, but still more high-quality RCTs are needed to support this conclusion. |
| 28 | Tang Q 2019 | 22 | 1987 | RCT | 2 | 3or4 | No | ER, VFSS, SSA, WST | Cochrane risk of bias tool | Acupuncture combined with rehabilitation training is effective for post stroke dysphagia and the combined effect is better than rehabilitation training alone. However, due to the small size and low quality of included RCTs, well designed RCTs with large-scale and high-quality are still required. |
| 29 | Feng Y 2019 | 32 | 3719 | RCT/q-RCT | 2 | 3,4 | Subcutaneous hemorrhage at local point, Postacupuncture hemorrhage, dizziness, nausea/vomiting | ER, AE, RR, IAP | GRADE, Cochrane risk of bias tool | Acupuncture and medicine combination was beneficial to the recovery of swallowing function after stroke, However, due to the lack of high-quality relevant literature, the evidence is still insufficient, large-scale and high-quality RCTs are needed. |
| 30 | Bi S 2019 | 54 | 4504 | RCT | 1,2 | 3,4 | No | ER, WST | CONSORT, STRICTA 5, Cochrane risk of bias tool | The efficacy and WST scores of acupuncture for post-stroke dysphagia were better than those of non-acupuncture therapies. But the quality of the RCT reports of acupuncture for post-stroke dysphagia included in this study was low, and future RCTs of acupuncture for post-stroke swallowing disorders should be designed and reported in strict accordance with CONSORT and STRICTA guidelines |
| 31 | Tang X 2019 | 16 | 1780 | RCT | 2 | 3 | Subcutaneous hemorrhage, Postacupuncture hemorrhage, pain | ER, WST, SSA | Cochrane risk of bias tool, Jadad score | Acupuncture combined with rehabilitation therapy was an effective treatment for post stroke dysphagia but still need more high quality RCTs to support this conclusion. |
| 32 | Zhao D 2019 | 28 | 2557 | RCT | 1,2 | 3,4 | Mentioned | ER, IAP, MBI, SWAL-QOL | Cochrane risk of bias tool | Acupuncture can effectively treat post-stroke dysphagia and improve patients' ability to perform activities of daily living and quality of life, However, the quality and quantity of literature included in the analysis were limited, further studies are needed to confirm this. |
| 33 | Huang J 2020 | 16 | 1216 | RCT | 2 | 3 | pain and hematoma | ER, WST, VFSS, IFRS, IAP, AE | Cochrane risk of bias tool | EA combined with SRT treatment for patients with PSD resulted in an additional benefit on the effective rate. Given the low quality of the included trials, these results are not conclusive, and further high-quality and large-scale RCTs are required to verify the therapeutic effects of EA on PSD. |
| 34 | Tan S 2020 | 12 | 903 | RCT | 2 | 3 | Postacupuncture hemorrhage, subcutaneous hemorrhage | ER, WST, VFSS | Cochrane risk of bias tool | Acupuncture combined with swallowing function training can effectively treat post-stroke dysphagia and promote the recovery of swallowing function. However, the included literatures have a certain publication bias, high inter -study heterogeneity, and the trial design is not rigorous enough, Therefore, more well-designed large sample clinical trials are still needed. |
| 35 | Lu Y 2021 | 39 | 3078 | RCT | 2 | 3 | No | WST, SSA, VFSS, SWAL-QOL, IFRS | Cochrane risk of bias tool | Acupuncture therapy can significantly improve the swallowing function of patients with dysphagia. But the evidence derived from these data was relatively weak. Follow-up studies are required to design more comprehensively the assessment of improvement in patients’ symptoms of dysphagia. |
| 36 | Zhong L 2021 | 35 | 3024 | RCT | 1、2 | 3 | Subcutaneous hemorrhage at local point, pain | SSA, IFRS, VFSS, WST | Cochrane risk of bias tool | Acupuncture for dysphagia after stroke has therapeutic efficacy and safety. More strict evaluation standards and high-quality RCT designs are necessary for further exploring acupuncture for the treatment of dysphagia after stroke. |
| 37 | Li T 2021 | 30 | 2446 | RCT | 1 | 3 | No | ER, significant efficiency, WST, SSA, IAP | Cochrane risk of bias tool | Electroacupuncture and acupuncture are clinically effective in the treatment of post-stroke dysphagia, but the study needs to be supported by more high-quality studies due to the limited number and quality of included studies. |
| 38 | Geng J 2021 | 18 | 1352 | RCT | 2 | 3 | Mentioned | ER, WST, VFSS, IFRS | Cochrane risk of bias tool | The clinical efficacy of conventional swallowing function training combined with EA for post-stroke swallowing dysfunction was better than that of swallowing function training alone, and there was no increase in adverse effects. |
| 39 | Wang J 2021 | 14 | 954 | RCT | 2 | 3 | No | ER, WST | Cochrane risk of bias tool | Acupuncture combined with swallowing training can promote the recovery of swallowing function in patients with post-stroke dysphagia, but more scientifically designed and high-quality randomized controlled trials are needed to validate the findings of this study. |
| 40 | Xia Y 2021 | 21 | 1532 | RCT | 1,2 | 3,4 | No | ER, SSA, WST | Cochrane risk of bias tool | Tongue three-needle therapy can improve the ER,WST and SSA score in patients with post-stroke dysphagia. Multi-center, high-quality and large-sample RCTs are still needed. |
| 41 | Cai G 2021 | 32 | 2665 | RCT | 1,2 | 3 | Subcutaneous hemorrhage, dizziness, Postacupuncture hemorrhage, pain | FIRS, SSA, ER, WST, VFSS, MBI, SWAL-QOL | GRADE, Cochrane risk of bias tool | Acupuncture is an effective and safe treatment for dysphagia, due to the lack of high-quality relevant literature, the evidence is still insufficient, further clinical trials are required to verify these results. |
| 42 | Hu S 2021 | 9 | 671 | RCT/q-RCT | 1,2 | 3 | No | ER, RR, WST, VFSS | Cochrane risk of bias tool | Tongue three-needle therapy can be an effective treatment for post-stroke dysphagia. However, the quality and quantity of literature included in the analysis were limited, and there is still a need for high-quality, large-sample, double-blind randomized controlled trials to provide a higher level of evidence. |
| 43 | Zhao N 2021 | 19 | 1874 | RCT | 1,2 | 3or4 | Subcutaneous hemorrhage, Postacupuncture hemorrhage | ER, SSA, SWAL-QOL | Cochrane risk of bias tool | The clinical efficacy and quality of life of patients with post-stroke dysphagia can be improved by using Xingnao Kaiqiao(brain awakening through opening orifices)acupuncture method alone or in combination with other therapies, but more high-quality, large-sample, multicenter RCTs are still needed. |
| 44 | Wang P 2022 | 7 | 637 | RCT | 1、2 | 3 | pain | KTST, SWAL QOL, SSA, NIHSS, RSST, MWST, FISES | Cochrane risk of bias tool | Acupuncture can be used as an effective treatment for dysphagia caused by pseudobulbar paralysis after stroke. Acupuncture combined with rehabilitation therapy has better effects. |
| 45 | Tang Y 2022 | 11 | 1069 | RCT | 2 | 3 | bleeding, hematomanand needle pain | ER, VFSS, SSA | Cochrane risk of bias tool; Jadad score | Nape acupuncture combined with rehabilitation training is more effective in the treatment of dysphagia after stroke than rehabilitation alone. However, we only have the strength of the medium and low quality to confirm this conclusion. In order to increase the strength, it is necessary to conduct multi-center and large-sample RTCs and update the research in time so that improve the strength of evidence. |
| 46 | Jiang H 2022 | 33 | 2680 | RCT | 1,2 | 3 | fainting needles, subcutaneous hemorrhage, pain, nausea, inappetence, | WST, SSA, VFSS, FISES, The rates of aspiration, The rates of aspiration pneumonia, DOSS, BI, SWAL-QOL, Duration of empty swallowing, Duration of 5 mL water swallowing | Cochrane risk of bias tool; Jadad score; GRADE | acupuncture or acupuncture combined with rehabilitation were better than using rehabilitation alone in the treatment of poststroke dysphagia. Meanwhile, multicenter RCTs with a large sample and a rigorous design are needed to explore whether acupuncture could replace other stimulative therapies in rehabilitation training. Moreover, acupoint combination, frequency, and the total number of treatments may be important factors that could influence therapeutic effect, which can provide guidance for subsequent similar RCTs. |
| 47 | Luo J 2023 | 10 | 517 | RCT | 2 | 3 | No | ER, SSA, VFSS | Cochrane risk of bias tool | Compared with balloon dilatation, acupuncture combined with balloon can significantly improve the swallowing function of patients, and it is also effective for patients of different courses, ages, and treatment course, while patients over 60 years old and the treatment course over 30 days may have better clinical outcomes.But the evidence strength was still low due to the high risk of bias in the included studies, and the example size was small. Therefore, more high-quality large-sample RCT literature analyses are urgently needed to confirm the conclusions. |
| 48 | Li L 2023 | 15 | 1094 | RCT | 1,2 | 3,4 | No | ER, WST, SSA | Cochrane risk of bias tool | The meta-analysis indicated that tongue acupuncture or tongue acupuncture combined with other therapies is clinically effective in the treatment of post-stroke dysphagia. However, there were some shortcomings in the literature included in this study. Because the evaluation of the methodological quality and quality of evidence is a subjective process and different researchers make independent judgments on each factor, the results of the studies may vary somewhat. In addition, acupuncture is difficult for the blind method in its implementation, and most experimental protocols are single-blind. In the future, randomized clinical studies with high quality, multi-center, large samples, and regular follow-up should be further carried out to improve the research quality. Strict design of the experimental scheme and adopting scientific research methods are also crucial to provide more meaningful evidence for the clinic. |
| 49 | Zuo T 2023 | 15 | 859 | RCT | 2 | 3 | No | ER, WST, SSA, PAS, FOIS, VFSS | PEDro,Cochrane risk of bias tool | Acupuncture combined with rTMS therapy were better than using rehabilitation alone in the treatment of post stroke dysphagia. Larger samples and higher quality RCTs are needed to further explore the efficacy of acupuncture combined with rTMS in the treatment of post stroke dysphagia. |
| 50 | Li X 2023 | 12 | 1358 | RCT | 2 | 3,3+4 | local numbness, subcutaneous hemorrhage, pain, papular dermatitis. | ER, SSA, PAS, VFSS, SWAL-QOL | Cochrane risk of bias tool,Jadad score,Grade Pro tool | EA is effective and safe in treating PSD. EA combined with conventional treatment or other interventions can significantly improve the clinical response rate and VFSS score in patients with PSD, without increasing the incidence of adverse reactions. There is no statistical difference between the two groups in terms of the improvement of SSA score, PAS score, and SWAL-QOL score. More high-quality RCT studies are still needed in the future to further explore the efficacy of EA in the treatment of PSD. |
| 51 | Sun H 2024 | 28 | 2879 | RCT | 1,2 | 3,4 | pain, ecchymosis, bleeding, gastrointestinal reaction | ER, WST, SSA, VFSS, SWAL-QOL | Cochrane risk of bias tool | Compared with control group, Xingnao Kaiqiao acupuncture group is more safe and effective in the treatment of PSD, which can effectively improve the quality of life of patients.Larger samples and higher quality RCTs are needed to confirm the conclusions. |

Notes: RCT: randomized controlled trial. q-RCT: quasi-RCT. SRs: systematic reviews. Intervention group: 1*=acupuncture/electroacupuncture alone. 2*=acupuncture/electroacupuncture combined with other therapies. Control group: 3*=rehabilitation/swallowing training, medicine, neuromuscular electrical stimulation, repetitive transcranial magnetic stimulation or baseline treatment. 4*=acupuncture on different points, sham acupuncture or no treatment. ADL: activities of daily living. AE: adverse events. BI: barthel index. CONSORT: consolidated standards of reporting trials. CSA: clinical symptoms assessment. CT7R:Caiteng 7 rank. DOSS: dysphagia outcome severity score.ER: efficacy rate. FDS: Fujishima Ichiro’ s dysphagia scale. FISES: Fujishima Ichiro swallowing Efficacy Score. GRADE: grading of recommendations assessment, development and evaluation. IA: individual activity. IAP: incidence of aspiration pneumonia. IFRS: Ichiro Fujishima rating scale. KSA: Kubota Toshio’s swallowing ability assessment. KTST: Kubota Toshio drinking water test. MAMC: mid-arm muscle circumference. MBI: modified barthel index. MNA: mini-nutritional assessment. MWST: modified water swallowing test. NIHSS: national institute of health stroke scale. PAS: penetration-aspiration scale. QOL: quality of life. RR: recovery rate. RSST: repeated saliva swallowing test. SFA: swallowing function assessment. SSA: standardized swallowing assessment. STRICTA: revised standards for reporting interventions in clinical trials of acupuncture. SWAL-QOL: swallowing-quality of life. TSF: triceps skinfold. VFSS: video fluoroscopic swallowing study.WST: water swallow test.

**references**

1. Wang, L. P., Jie, Y. (2006). Systematic evaluation of acupuncture and moxibustion of dysphagia after stroke. Chinese Acupuncture & Moxibustion, 26, 141–146.

2. Xie, Y., Wang, L., He, J., Wu, T. (2008). Acupuncture for dysphagia in acute stroke. Cochrane Database Syst Rev, CD006076. doi: 10.1002/14651858. CD006076. Pub2.

3. Zhao, X. (2010) Acupuncture therapy combined with swallowing function training for dysphagia after acute stroke: a systematic review. [dissertation/master's thesis]. [China (Sichuan)]: Chengdu University of TCM

4. Yuan, M., Yang, Z., Guo, J., Zhang, X., Zheng, Q. (2011). Meta-analysis of the clinical effect of acupuncture combined with rehabilitation training on dysphagia after stroke. Chinese Journal of Rehabilitation Medicine 26, 467-470. doi: 10.3969/j.issn.1001-1242.2011.05.017.

5. Li, S., Fu, L. X., Huang, X. D., Zhao, R. (2011). Systematic review of acupuncture for treatment of pseudobulbar palsy due to wind stroke in clinical studies. Journal of Clinical Acupuncture and Moxibustion 27, 1-6. doi: 10.3969/j.issn.1005-0779.2011.01.001.

6. Long, Y., and Wu, X. (2012). A meta-analysis of the efficacy of acupuncture in treating dysphagia in patients with a stroke. Acupunct Med 30, 291-7. doi: 10.1136/acupmed-2012-010155

7. Zhu, Y., Fu, L., Li, S., Pan, W. (2012). Systematic review of acupuncture and moxibustion for dysphagia caused by post-stroke pseudobulbar palsy. Shandong Journal of Traditional Chinese Medicine 31, 741-745. doi: 10.16295/j.cnki.0257-358x.2012.10.021.

8. Chen, J., Liang, W., Liu, Q., Qin, S., Hei, H. (2015). Effectiveness and safety of resuscitation-inducing acupuncture for post-stroke dysphasia: A systematic review. Journal of Guangzhou University of Traditional Chinese Medicine, 607-614. doi: 10.13359 / j.carol carroll nki GZXBTCM. 2015.04.006

9. Hu, T., Wang, X., Yu, J., Chen, G., He, Y. (2015). A Meta-analysis of the Therapeutic Effect of Nape Acupuncture on Post-stroke dysphagia in Domestic Literature. Shanghai Journal of Acupuncture and Moxibustion, 1250-1254. doi: 10.13460/j.issn.1005-0957.2015.12.1250

10. Xiang, Y. (2015) Acupuncture for dysphagia after stroke: a systematic review. [dissertation/master's thesis]. [China (Sichuan)]: Chengdu University of TCM

11. Yu, C., Shen, B., Xu, S. (2016). Systematic Review of Acupuncture-moxibustion for Deglutition Disorders after Cerebral Stroke. Shanghai Journal of Acupuncture and Moxibustion 35, 1126–1129. doi: 10.13460/j.issn.1005-0957.2016.09.1126

12. Meng, D., Shang, Y., Fu, Y., Wang, X., Xu, C., Tan, Y., et al. (2016). Clinical Literature Study of Acupuncture and Moxibustion in the Treatment of Post -stroke Dysphagia based on Meta Analysis. Chinese Medicine Modern Distance Education of China 14, 148-150, p.3-p.4. doi: 10.3969/j.issn.1672-2779.2016.16.064.

13. Liu, H. (2016) Acupuncture for poststroke dysphagia: A Systematic Review. [dissertation/master's thesis]. [China (Sichuan)]: Chengdu University of TCM

14. Ye, Q., Xie, Y., Shi, J., Xu, Z., Ou, A., and Xu, N. (2017). Systematic Review on Acupuncture for Treatment of Dysphagia after Stroke. Evid Based Complement Alternat Med 2017, 6421852. doi: 10.1155/2017/6421852

15. Zhang, Y., Zhang, X., Xiong, Y., Chen, Z., Du, F., Shen, J., et al. (2017). Meta-analysis of Randomized Controlled Trials on Electro-acupuncture in the Treatment of Dysphagia after Stroke. Journal of Clinical Acupuncture and Moxibustion 33, 75-79. doi: 10.3969/j.issn.1005-0779.2017.08.022

16. Zhang, S., Zeng, X., Wang, Y., Wang, Y., Zhang, T., Luo, X., et al. (2017). (in Chinese). A Systematic Review of Randomized Controlled Clinical Trials of Electro-acupuncture Treatment of dysphagia after stroke. China Health Care & Nutrition 27, 5-7. doi: 10.3969/j.issn.1004-7484.2017.04.005

17. Wang, C., Chai, T., Wang, W., Xiang, Q. (2017). The Efficacy of Acupuncture for Post-stroke Dysphagia: A Systematic Review and Meta-analysis. Guiding Journal of Traditional Chinese Medicine and Pharmacy 23, 72-76. doi: 10.13862 / j.carol carroll nki cn43-1446 / r. 2017.19.026

18. Xu, M. (2017). Meta-analysis of curative effects of acupuncture therapy on dysphagia after stroke. Journal of Medical Science Yanbian University 40, 113-115. doi: 10.16068/j.1000-1824.2017.02.011

19. Liao, M., Huang, L., Zeng, Z., He, J. (2017). (in Chinese). Meta-analysis of Jin's three-needle in the treatment of post-stroke pseudobulbar palsy. Lishizhen Medicine and Materia Medica Research 28, 3023-3030. doi: 10.3969/j.issn.1008-0805.2017.12.076

20. Li, L. X., Deng, K., Qu, Y. (2018). Acupuncture Treatment for Post-Stroke Dysphagia: An Update Meta-Analysis of Randomized Controlled Trials. Chin. J. Integr. Med. 24, 686–695. doi: 10.1007/s11655-018-3005-3

21. Shi, L. (2018). Acupuncture Combined with Rehabilitation Training in Treatment of Post-Stroke Dysphagia: An Evidence-Based Medicine Study. Journal of Clinical Acupuncture and Moxibustion 34, 62-66. doi: 10.3969/j.issn.1005-0779.2018.07.017

22. Yang, J., Wang, C. (2018). Meta analysis of therapeutic effect of tongue tri-points therapy on post stroke pseudobulbar palsy. Chinese Journal of Acupuncture and Moxibustion (Electronic Edition) 7, 116-120. doi: 10.3877/cma.j.issn.2095-3240.2018.03.009

23. Wang, Y. (2018) Systematic evaluation of clinical efficacy of acupuncture combined rehabilitation training in treating dysphagia after stroke. [dissertation/master's thesis]. [China (Shanxi)]: Shanxi University of Chinese Medicine

24. Chen, Y. (2018) Meta-analysis of acupuncture treatment for dysphagia after stroke. [dissertation/master's thesis]. [China (Changchun)]: Changchun University of Chinese Medicine

25. Li, L., Deng, K. (2019). Acupuncture combined with swallowing training for poststroke dysphagia: a meta-analysis of randomised controlled trials. Acupunct Med 37, 81–90. doi: 10.1136/acupmed-2016-011305

26. Wang, H., Zhou, H., Li, C., Zhang, Z., Liu, J. (2019). Meta-Analysis of Acupuncture Combined with Ｒehabilitation Training for Post-Stroke Dysphagia. Journal of Clinical Acupuncture and Moxibustion 35, 75-81. doi: 10.3969/j.issn.1005-0779.2019.10.020

27. Xu, L., Zhang X., Pu, T.., Luo, S., Luo, L., Huang, L., et al. (2019). Consciousness Restoring for Resuscitation Acupuncture Therapy for Dysphagia after Stroke: A Systematic Review and Meta-Analysis.Journal of Yunnan University of Chinese Medicine 42, 53-60, doi: 10.19288 / j.carol carroll nki. Issn 1000-2723.2019.03.010

28. Tang, Q., Zhao, X., Zhu, L. (2019). Systematic evaluation and meta-analysis of the effect of acupuncture combined with rehabilitation training on dysphagia after stroke. West China Medical Journal 34, 531-538. doi: 10.7507/1002-0179.201903221

29. Feng, Y. (2019) Syndrom types, treatment and systematic review of the curative effect of dysphagia after stroke. [dissertation/master's thesis]. [China (Shandong)]: Shandong University of Traditional Chinese Medicine

30. Bi, S. (2019) Efficacy of acupuncture and moxibustion randomized controlled trial in the treatment of dysphagia after stroke: a meta-analysis. [dissertation/master's thesis]. [China (Guangzhou)]: Guangzhou University of Chinese Medicine

31. Tang, X., Wang, L., Huang, P., Lan, J., Xu, N. (2019). Meta-analysis of Acupuncture Combined with Rehabilitation Therapy for Treatment of Dysphagia After Stroke.Journal of guangzhou university of Traditional Chinese Medicine 36, 514-520. doi: 10.13359 / j.carol carroll nki GZXBTCM. 2019.04.015

32. Zhao, D., Zou, Y., Zhang, H., Guo, K., Chen, M., Qiu, R., et al. (2019). Systematic Evaluation of Clinical Efficacy of Acupuncture in the Treatment of Post-stroke Dysphagia. Journal of Hunan University of Chinese Medicine 39, 986-993. doi: 10.3969 / j. issn.1674 - 070 x.2019.08.013

33. Huang, J., Yao, S. Qin, X., Shen, M., Wu, M., Huang, Y. (2020). Clinical Effects and Safety of Electroacupuncture for the Treatment of Post stroke Dysphagia: A Comprehensive Systematic Review and Meta-Analysis. Evid Based Complement Alternat Med 2020, 1560978. doi: 10.1155/2020/1560978

34. Tan, S., Liao, J., Zhang, L., Liu, Y., Bai, W. (2020). Meta analysis of acupuncture combined with swallowing function training in the treatment of dysphagia after stroke. China Medical Herald 17, 98-102. doi: 1673-7210 (2020) 05(a)-0098-05

35. Lu, Y., Chen, Y., Huang, D., Li, J. (2021). Efficacy of acupuncture for dysphagia after stroke: a systematic review and meta-analysis. Ann Palliat Med 10, 3410–3422. doi: 10.21037/apm-21-499

36. Zhong, L., Wang, J., Li, F., Bao, X., Liu, H., Wang, P. (2021). The Effectiveness of Acupuncture for Dysphagia after Stroke: A Systematic Review and Meta-Analysis. Evid Based Complement Alternat Med 2021, 8837625. doi: 10.1155/2021/8837625

37. Li, T., Feng, L., Sun, Q., Xie, X., Wang, B., Li, C., et al. (2021). (in Chinese). Meta-analysis and GRADE evidence level evaluation of randomized controlled trials of acupuncture and electroacupuncture for post-stroke dysphagia. World Journal of Integrated Chinese and Western Medicine 16, 804-811,816. doi: 10.13935/j

38. Geng, J., Gao, Y., Cao, B. Zhao, Y., Liu, W., Wang, J. (2021). Meta-analysis of the effect of electroacupuncture on post-stroke dysphagia. Shaanxi Journal of Traditional Chinese Medicine 42, 1814-1818. doi: 10.3969/j.issn.1000-7369.2021.12.042

39. Wang, J., Xiao, S., Wen, T., Liu, H. (2021). Meta-analysis of rehabilitation effect of acupuncture and swallowing training on dysphagia after stroke. China Medicine and Pharmacy 11, 71-76. doi: 10.3969/j.issn.2095-0616.2021.24.018

40. Xia, Y., Huang, H., Jiang, D., Huang, F., Yi, W. (2021). A systematic review and sequential analysis of trials of tongue three-needle therapy for post-stroke dysphagia. Journal of guangzhou university of Chinese medicine, 38, 1370-1376. doi: 10.13359 / j.carol carroll nki GZXBTCM. 2021.07.013.

41. Cai, G. (2021) Acupuncture treatment of dysphagia: Systematic review. [dissertation/master's thesis]. [China (Heilongjiang)]: Heilongjiang University of Chinese Medicine

42. Hu, S., Shi, L. (2021). A meta-analysis of the clinical efficacy of tongue three-needle in the treatment of post-stroke dysphagia. Hunan Journal of Traditional Chinese Medicine 37, 126-129. doi: 10. 16808 / j. cnki. issn1003-7705. 2021. 01. 049

43. Zhao, N., Li, J., Zhang, Y. (2021). Meta-analysis of Xingnao Kaiqiao acupuncture for post-stroke dysphagia. Chinese Journal of Acupuncture and Moxibustion (Electronic Edition) 10, 165-170. doi: 10.3877 /cma. j. issn. 2095-3240. 2021. 04. 008

44. Wang, P., Ma, X., Huang, J., Li, J., Ma, L., Xu, D., et al. (2022). Effect of acupuncture treatment on dysphagia caused by pseudobulbar paralysis after stroke: a systematic review and meta-analysis. Ann Palliat Med. doi: 10.21037/apm-21-3551.

45. Tang, Y., Liang, R., Gao, W., Zhang, S., Liang, B., Zhu, L. (2022). A meta-analysis of the effect of nape acupuncture combined with rehabilitation training in the treatment of dysphagia after stroke. Medicine (Baltimore) 101, e31906. doi: 10.1097/MD.0000000000031906.

46. Jiang, H., Zhang, Q., Zhao, Q., Chen, H., Nan, X., Liu, M., et al. (2022). Manual Acupuncture or Combination of Rehabilitation Therapy to Treat Poststroke Dysphagia: A Systematic Review and Meta-Analysis of Randomized Controlled Trials. Evidence-Based Complementary and Alternative Medicine, 8803507–8803507.

47. Luo, J., Huang, B., Zheng, H., Yang, Z., Xu, M., Xu, Z., et al. (2023). Acupuncture combined with balloon dilation for post-stroke cricopharyngeal achalasia: A meta-analysis of randomized controlled trials. Front Neurosci 16, 1092443. doi: 10.3389/fnins.2022.1092443.

48. Li, L., Xu, F., Yang, S., Kuang, P., Ding, H., Huang, M., et al. (2023). Tongue acupuncture for the treatment of post-stroke dysphagia: a meta-analysis of randomized controlled trials. Front Neurosci 17, 1124064. doi: 10.3389/fnins.2023.1124064.

49. Zuo, T. (2023). Meta-analysis of acupuncture combined with repetitive transcranial magnetic stimulation in the treatment of dysphagia after stroke. Hunan Journal of Traditional Chinese Medicine 39, 122–127. doi: 10.16808/j.cnki.issn1003-7705.2023.07.033.

50. Li, X., Lu, L., Fu, X., Li, H., Yang, W., Guo, H., et al. (2023). Systematic review and meta-analysis of the efficacy and safety of electroacupuncture for poststroke dysphagia. Front Neurol 14, 1270624. doi: 10.3389/fneur.2023.1270624.

51. Sun, H., Yao, Q., Xiong, L., Jiang, S. (2024). Meta-analysis of Efficacy and Safety Evaluation of Xingnao Kaiqiao Needle in the Treatment of Dysphagia in Stroke Patients. Chinese Journal of Ethnomedicine and Ethnopharmacy 33, 103–110. doi: 10.3969/j.issn.1007-8517.2024.05.zgmzmjyyzz202405022.
